# Uracil DNA Glycosylase Counteracts APOBEC3G-Induced Hypermutation of Hepatitis B Viral Genomes: Excision Repair of Covalently Closed Circular DNA

**DOI:** 10.1371/journal.ppat.1003361

**Published:** 2013-05-16

**Authors:** Kouichi Kitamura, Zhe Wang, Sajeda Chowdhury, Miyuki Simadu, Miki Koura, Masamichi Muramatsu

**Affiliations:** Department of Molecular Genetics, Kanazawa University Graduate School of Medical Science, Kanazawa, Japan; University of California San Diego, United States of America

## Abstract

The covalently closed circular DNA (cccDNA) of the hepatitis B virus (HBV) plays an essential role in chronic hepatitis. The cellular repair system is proposed to convert cytoplasmic nucleocapsid (NC) DNA (partially double-stranded DNA) into cccDNA in the nucleus. Recently, antiviral cytidine deaminases, AID/APOBEC proteins, were shown to generate uracil residues in the NC-DNA through deamination, resulting in cytidine-to-uracil (C-to-U) hypermutation of the viral genome. We investigated whether uracil residues in hepadnavirus DNA were excised by uracil-DNA glycosylase (UNG), a host factor for base excision repair (BER). When UNG activity was inhibited by the expression of the UNG inhibitory protein (UGI), hypermutation of NC-DNA induced by either APOBEC3G or interferon treatment was enhanced in a human hepatocyte cell line. To assess the effect of UNG on the cccDNA viral intermediate, we used the duck HBV (DHBV) replication model. Sequence analyses of DHBV DNAs showed that cccDNA accumulated G-to-A or C-to-T mutations in APOBEC3G-expressing cells, and this was extensively enhanced by UNG inhibition. The cccDNA hypermutation generated many premature stop codons in the P gene. UNG inhibition also enhanced the APOBEC3G-mediated suppression of viral replication, including reduction of NC-DNA, pre-C mRNA, and secreted viral particle-associated DNA in prolonged culture. Enhancement of APOBEC3G-mediated suppression by UNG inhibition was not observed when the catalytic site of APOBEC3G was mutated. Transfection experiments of recloned cccDNAs revealed that the combination of UNG inhibition and APOBEC3G expression reduced the replication ability of cccDNA. Taken together, these data indicate that UNG excises uracil residues from the viral genome during or after cccDNA formation in the nucleus and imply that BER pathway activities decrease the antiviral effect of APOBEC3-mediated hypermutation.

## Author Summary

Human cytidine deaminases, AID/APOBECs, are restriction factors against various types of viruses. These proteins have the ability to introduce a cytidine-to-uridine (C-to-U) hypermutation in the viral DNAs of the hepadnaviruses hepatitis B virus (HBV) and duck HBV (DHBV) models. It is well known that uracil residues in human genomic DNA are removed by uracil-DNA glycosylase (UNG), resulting in the creation of abasic sites that are repaired by downstream repair factors. However, the consequence of uracil removal from the viral genomic DNA remains controversial, given that it may be possible for abasic sites to trigger DNA degradation with strand breakage. We investigated the role of UNG in viral hypermutation and hepadnaviruses replication using *in vitro* cell culture systems. We found that UNG inhibition enhanced APOBEC3G-induced hypermutation of hepadnaviral DNAs, especially DHBV cccDNA, a template used for viral replication in the nucleus. We measured the replication ability of purified cccDNA and found that recloned cccDNA from cells expressed by both APOBEC3G and UNG inhibitor protein replicated less efficiently due to higher hypermutation rates. These results suggest that hepadnaviruses usurp the repair system of host cells to compete with AID/APOBEC mutators.

## Introduction

The hepatitis B virus (HBV) is one of the major causative factors of liver cirrhosis and hepatocellular carcinoma. Chronic inflammation due to persistent HBV infection plays a major causative role in these severe liver diseases. However, it is still unknown how HBV establishes persistent infection and how this infection results in these diseases [1], [2]. The HBV genome in virions forms a relaxed circular DNA (rcDNA) that is converted into covalently closed circular DNA (cccDNA) in the nuclei of infected hepatocytes. The cccDNA transcribes all viral RNAs including pregenomic (pg) RNA as a replicative RNA intermediate. In the cytoplasm, pgRNA, viral core, and polymerase proteins are assembled into the nucleocapsid (NC), after which the pgRNA is converted into an rcDNA by viral polymerase activity. The mature NCs are transferred to either the endoplasmic reticulum to be secreted after combining with envelope proteins or the nucleus to form cccDNA again for the next replication cycle. Although the host repair system is thought to play a major role in conversion of rcDNA into cccDNA, the molecules responsible for the conversion have not been determined experimentally [3], [4], [5].

cccDNA plays a key role in the persistence of viral infection because it is maintained as a stable episome in the nucleus. Moreover, cccDNA is not targeted by anti-HBV drugs and thus enables the re-establishment of viral replication after cessation of antiviral therapy [4], [6], [7]. Despite the importance of cccDNA in HBV chronic infection, host factors that control cccDNA are poorly understood in the absence of an efficient experimental system that can produce HBV cccDNA at a level sufficient for analysis. In view of the limitation of HBV *in vitro* systems, the duck HBV (DHBV) model has been commonly used to study HBV infection [8]. DHBV is an avian counterpart of HBV, sharing fundamental features including genomic organization, replication processes, and biological characteristics [9]. Importantly, DHBV produces cccDNA more efficiently than HBV [10].

Previously, we isolated a B-cell-specific gene, activation-induced cytidine deaminase (AID), which is essential for class-switch recombination and somatic hypermutation of immunoglobulin genes [11], [12]. AID belongs to the APOBEC (apolipoprotein B mRNA editing catalytic polypeptide) family of proteins. In humans, this family comprises at least 11 members, including AID and APOBECs 1, 2, 3A, 3B, 3C, 3DE, 3F, 3G, 3H, and 4. These AID/APOBEC proteins have enzyme activity that can deaminate a cytidine base in DNA and/or RNA and thereby generate a uridine base. APOBEC3G (A3G) restricts replication of retroviruses, including human immunodeficiency virus type 1 (HIV-1), and retrotransposable elements [13], [14], [15]. A3G has been shown to also restrict other viruses such as HBV [16], [17], [18], [19], [20], [21], [22], [23], [24], but the exact mechanism of restriction of HBV replication remains unresolved. Earlier studies suggested that accumulation of extensive G-to-A (in an opposite strand of C-to-U) hypermutation in retroviral DNA might initiate the deamination-mediated restriction pathway of A3G [25], [26]. However, such hypermutation may not account for rapid reduction of HBV NC-DNA by A3G overexpression because only a limited fraction of NC-DNA accumulates these extensive mutations [16], [19], [27], [28]. Another proposed mechanism is that A3G is encapsidated within NC with pgRNA and interferes in the process of minus-stranded DNA synthesis, such that a catalytically inactive mutant of A3G has been shown to still inhibit viral replication [19], [21]. Deaminase-independent restriction by A3G has also been demonstrated for HIV-1 [14], [15].

Human uracil DNA glycosylase (UNG) is a base excision repair (BER) enzyme that removes uracil residues from DNA following dUTP misincorporations or cytosine deaminations [29]. UNG is also essential for class-switch recombination and somatic hypermutation. In UNG deficiency (human and mouse), class-switch recombination is markedly reduced because uracil bases generated by AID do not produce DNA strand breaks in the absence of UNG. However, UNG-deficient mice and patients accumulate more frequent C-to-T and G-to-A somatic hypermutations because uracil bases generated by AID remain as thymine residues in UNG-deficient condition [29], [30]. The UNG gene encodes 2 alternative splicing isoforms with unique N-terminal amino acid sequences, mitochondrial type UNG1 and nuclear type UNG2. Early HIV-1 studies showed that UNG2 is encapsidated into the virion through physical association with the Vpr protein and reduces the mutation rate of viral DNA [31], [32]. However, the contribution of UNG activity to APOBEC3 (A3)-mediated HIV-1 restriction is controversial. Yang *et al.* proposed that uracil residues generated by A3G might be eliminated by UNG associated with Vpr and that subsequent DNA cleavage of abasic sites by apurinic/apyrimidinic endonuclease-1 (APE-1) might occur [33]. Meanwhile, other groups reported that A3G-mediated HIV-1 restriction occurs even in the absence of UNG [34], [35], [36]. Importantly, HBV does not harbor the Vpr counterpart, and UNG encapsidation in HBV particles has not been reported. Unlike HIV-1, HBV forms episomal cccDNA, which is a potential target for nuclear UNG activity; however, whether APOBECs can hypermutate the cccDNA and whether UNG has any role in cccDNA maintenance has not been investigated.

In the present study, we investigated the possible role of UNG in A3G-mediated antiviral activities on HBV and DHBV. When UNG activity was inhibited by expression of the UNG inhibitory protein (UGI), hypermutation of HBV and DHBV NC-DNA was enhanced in A3G-expressing hepatocytes. We found that more than half of DHBV cccDNA clones accumulated extensive hypermutation by A3G overexpression and UNG inhibition. Moreover, we demonstrated that the cccDNA isolated from cells expressing both A3G and UGI showed decrease in replication activity. These experimental observations indicate that UNG efficiently repairs dysfunctional C-to-U mutations induced by A3G in cccDNA.

## Results

### UNG inhibition enhanced A3G-induced hypermutation of HBV NC-associated DNA

**Figure 1:**
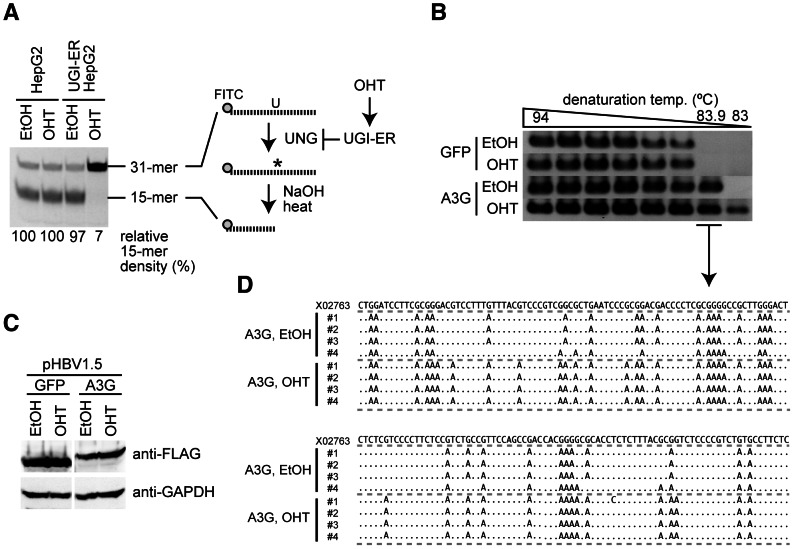
UNG inhibition enhances A3G-induced hypermutation of HBV NC-associated DNA. (A) Uracil excision activity was measured using an UNG assay with a synthetic single-stranded DNA containing a dU. UGI–ER-expressing HepG2 and parental cells were treated with either 1 µM OHT or the same amount of solvent (ethanol, EtOH) for 48 h, and UNG activity was measured. Asterisk (*), abasic site. The percent density of signals for the 15-mer is indicated. Signal density of the OHT-treated HepG2 cells was defined as 100%. (B) The HBV replicon plasmid (pHBV1.5) and FLAG-A3G or FLAG-GFP expression vectors were used to transfect the UGI–ER HepG2 cells. Twenty-four hours after transfection, 1 µM OHT (or EtOH) was added to activate the UGI–ER protein. After further 48-h incubation, the cells were harvested and the HBV NC-DNA was purified. HBV DNAs from each transfectant were amplified by 3D-PCR with denaturation temperature gradient of 94–83°C. (C) Expression of exogenous A3G and GFP proteins in the conditions of (B) was determined by Western blotting with anti-FLAG antibody. Expression of GAPDH is also shown to demonstrate equivalent protein loading. (D) Alignment of hypermutated HBV sequences. PCR fragments from the 83.9°C denaturation temperature reaction in (B) were excised from agarose gel and cloned into T vectors, and then 4 randomly selected clones from each sample were sequenced. The sequence (GenBank accession number: X02763) from the pHBV1.5 is shown on the top as a reference. Dots in the alignment represent identity to the reference sequence.

To investigate the potential role of UNG in HBV hypermutation, the UNG activity of the human hepatocyte cell line HepG2 was suppressed with UGI, which is an irreversible inhibitor that forms an exceptionally stable complex with the UNG protein [37]. We generated the HepG2 cell line that stably expressed the UGI–estrogen receptor (ER) protein by retrovirus-mediated gene transduction following drug selection. We had previously demonstrated that the addition of an ER ligand, 4-hydroxytamoxifen (OHT), to the culture medium activated the enzymatic activity of the fusion partner [38], [39]. Accordingly, we reasoned that expression of the UGI–ER fusion protein could be applied to control UNG activity in HBV-replicating cells. The UNG assay revealed that OHT stimulation of the UGI–ER protein resulted in very limited UNG activity, amounting to 7% of the activity in the parental HepG2 cells, whereas 97% of activity remained in unstimulated (EtOH) cells (Figure 1A). Using this cell line, we estimated the effects of UNG inhibition on A3G-induced hypermutation of HBV NC-DNA. An HBV replicon plasmid, pHBV1.5 [40], [41], was cotransfected with the FLAG-tagged A3G expression plasmid [or green fluorescent protein (GFP) as a negative control] into the UGI–ER HepG2 cells. Three days after transfection, the cells were harvested and cytoplasmic NCs were purified. The NC-DNA was analyzed by differential DNA denaturation polymerase chain reaction (3D-PCR) on a region of the X gene [28]. The 3D-PCR technique is a highly sensitive assay for detecting AT-rich DNA. It was applied to reveal the presence of hypermutation in NC-DNA. Consistent with reports from other groups [18], [19], [23], [24], [28], [42], [43], A3G expression induced HBV hypermutation, represented as a lower denaturation temperature band (83.9°C) than negative controls in 3D-PCR (Figure 1B). OHT addition enhanced the hypermutation in the A3G-expressing cells because the band was detected at the lowest melting temperature (83.0°C). OHT addition did not influence A3G transgene expression in these cells (Figure 1C). The 83.9°C PCR products shown in Figure 1B were cloned and sequenced. As predicted by the 3D-PCR assay, the cloned PCR fragments accumulated extensive G-to-A mutations (Figure 1D).

We also suppressed UNG activity using a short-interfering RNA (siRNA) approach to avoid any artifacts due to the UGI–ER inducible activation system. We used 293T cells for the siRNA experiment because of better transfection efficiency of the siRNA than that afforded by HepG2 cells. For viral replication in human embryonic kidney 293T cells, another replicon plasmid pPB that expresses HBV pgRNA by the CMV promoter was used [44], [45]. The UNG assay revealed that both UNG-specific siRNAs reduced UNG activity in 293T cells, although at low suppression efficiency (a maximum of 47% of the control; Figure S1A). Nonetheless, 3D-PCR showed amplification at a slightly lower denaturation temperature, indicating the presence of hypermutated HBV DNA from the UNG-specific siRNA-treated cells (Figure S1B). These data indicate that inhibition of UNG activity increases A3G-induced hypermutation of HBV NC-DNA.

### UNG inhibition enhanced endogenous deaminase-induced hypermutation of HBV NC-associated DNA

**Figure 2:**
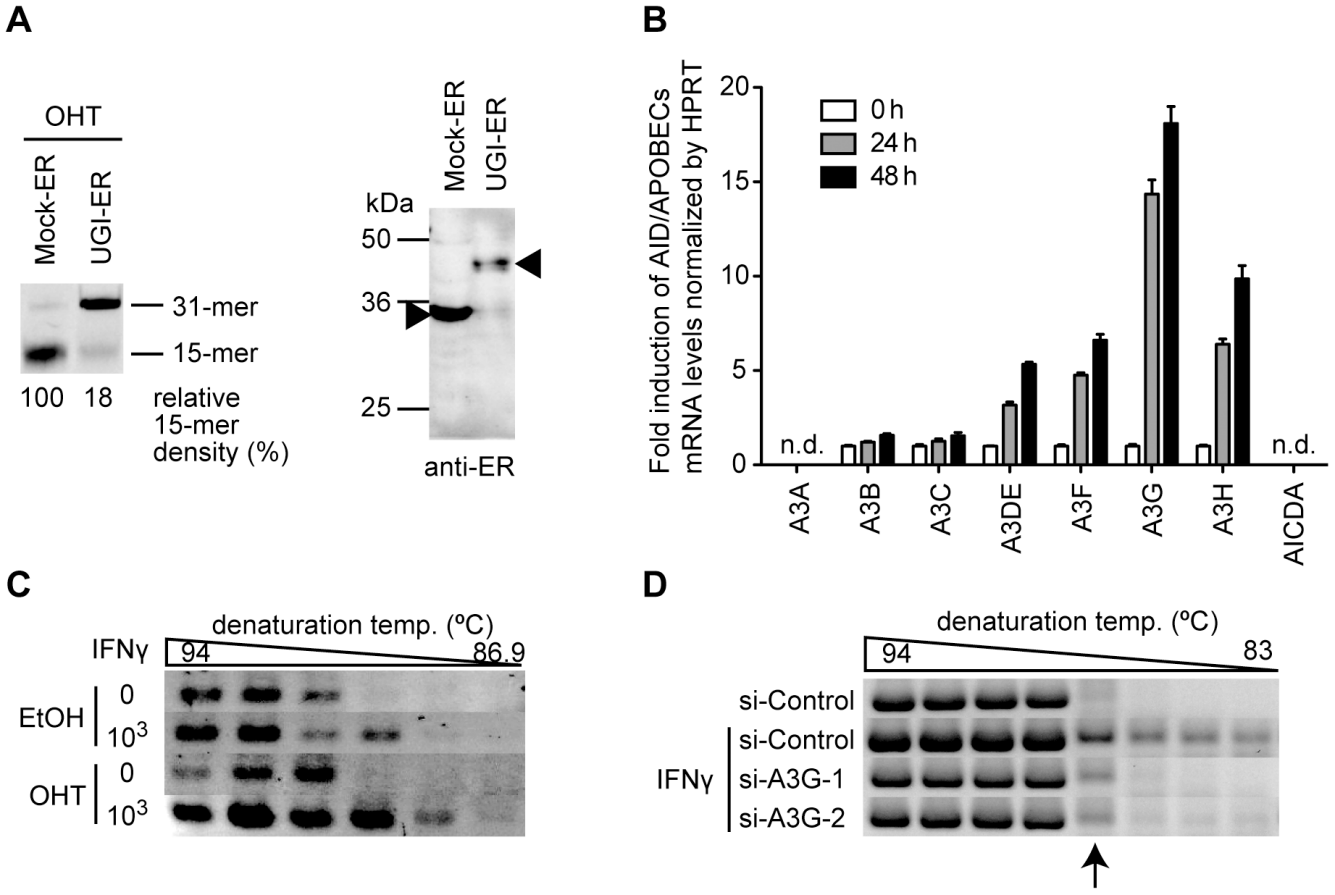
UNG inhibition enhances the endogenous deaminase-induced hypermutation of HBV NC-associated DNA. (A) Uracil excision activity in a HepG2 double-stable transfectant of UGI–ER (or Mock–ER) and HBV was assessed as in Figure 1A (left panel). Expression of UGI–ER and mock–ER proteins was confirmed by Western blotting with anti-ER antibody (right panel, indicated by arrowheads). (B) Quantification of the expression of AID/APOBECs on interferon stimulation. HepG2 cells were stimulated with 1000 U/mL IFNγ for 24 or 48 h. qRT-PCR was performed to determine the expression level for each deaminase. Under these conditions, AID (AICDA) and A3A expression were not detected (n.d.). Expression at 0 h was defined as a 1-fold change. (C) 3D-PCR analysis of the stable double transfectants of UGI–ER and HBV used in (A). The cells were treated with 1000 U/mL IFNγ and 1 µM OHT as indicated. After the 48-h incubation, the cells were harvested and the HBV NC-DNA was analyzed. (D) 3D-PCR analysis of the IFNγ-stimulated and A3G knockdown cells. The A3G-specific (or control) siRNAs and pPB were used to transfect HepG2 cells, and at 16 h after transfection, the cells were treated with 1000 U/mL IFNγ. After further 48-h incubation, the HBV NC-DNA was purified and analyzed. PCR fragments (87.2°C) indicated by an arrow were sequenced and the results were shown in Figure S1D.

Next, we investigated whether the enhancement of hypermutation by UNG inhibition was reproduced by endogenous AID/APOBEC3 proteins. We generated a stable HepG2 cell line that constitutively supports both HBV replication and UGI–ER expression in order to establish a transfection-free system (see Materials & Methods for details). Inhibition of UNG activity by OHT addition in this cell line was confirmed by the UNG assay (Figure 2A). IFNγ was used to stimulate the cells to induce endogenous APOBEC deaminases, and changes in deaminase gene expression levels were measured by quantitative reverse transcription-PCR (qRT-PCR). Consistent with that in previous studies [20], [46], [47], A3G was the major responder to IFNγ stimulation among AID/APOBEC3s (A3s) in this cell line (Figure 2B). The hypermutation load on NC-DNA in IFNγ-stimulated UGI–ER HepG2 was analyzed by 3D-PCR. Our analyses revealed that UNG inhibition enhanced IFNγ-induced NC-DNA hypermutation (Figure 2C). To evaluate the contributions of endogenous APOBEC3G, we used A3G-specific siRNAs. The efficiency of siRNA knockdown was determined by qRT-PCR (Figure S1C). As shown in Figure 2D, the knockdown of A3G expression counteracted the induction of hypermutation by IFNγ, suggesting that A3G is responsible for HBV hypermutation induced by IFNγ stimulation. The 87.2°C PCR products shown in Figure 1D were cloned and sequenced, and confirmed the hypermutation (Figure S1D). These data suggest that UNG counteracts the deamination of HBV NC-DNA triggered by endogenous A3s.

### Most HBV NC-associated DNAs were not affected by UNG inhibition

**Figure 3:**
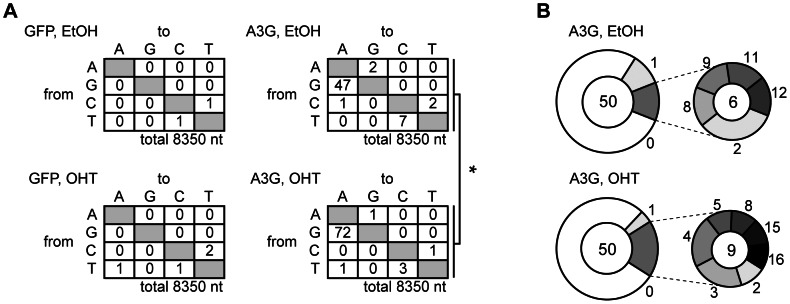
Mutation frequency of HBV NC-associated DNA in A3G expression and UNG inhibition. DNA fragments amplified through standard PCR (94°C in Figure 1B) were cloned into T vectors. NC-DNA sequences from randomly selected 50 clones were analyzed for each group. (A) Mutation matrices of the HBV NC-DNA with or without A3G expression and in the presence or absence of OHT. *P<0.05. The statistical significance for the frequency of G-to-A mutations was calculated by chi-square test. (B) Pie charts represent the proportion of clones with G-to-A and C-to-T mutations for (A). The total number of sequenced clones is indicated in the center. The number of mutations is indicated on the periphery of the pie segment. Thirty-nine clones were intact in both samples (white segment, 0 mutation). The satellite chart depicts the proportion of clones with more than 2 mutations.

To determine the overall hypermutation frequency of HBV DNA, we sequenced the NC-DNA from the A3G-transfected UGI–ER HepG2. PCR fragments of the X gene partial sequence (94°C for denaturation) of NC-DNA were cloned in a T vector. Fifty clones of each sample were randomly selected for DNA sequencing. Figure 3A shows the mutations found in the sequenced clones. Consistent with 3D-PCR results, the total G-to-A mutation frequency was enhanced by UNG inhibition in A3G transfectants (indicated as “A3G, OHT” in Figure 3). However, 39 of the 50 sequenced clones were free from hypermutation in both UNG-inhibited and uninhibited cells (Figure 3B). Previous studies [16], [19], [27], [28] also reported few clones harboring A3G-induced mutations in NC-DNA.

**Figure 4:**
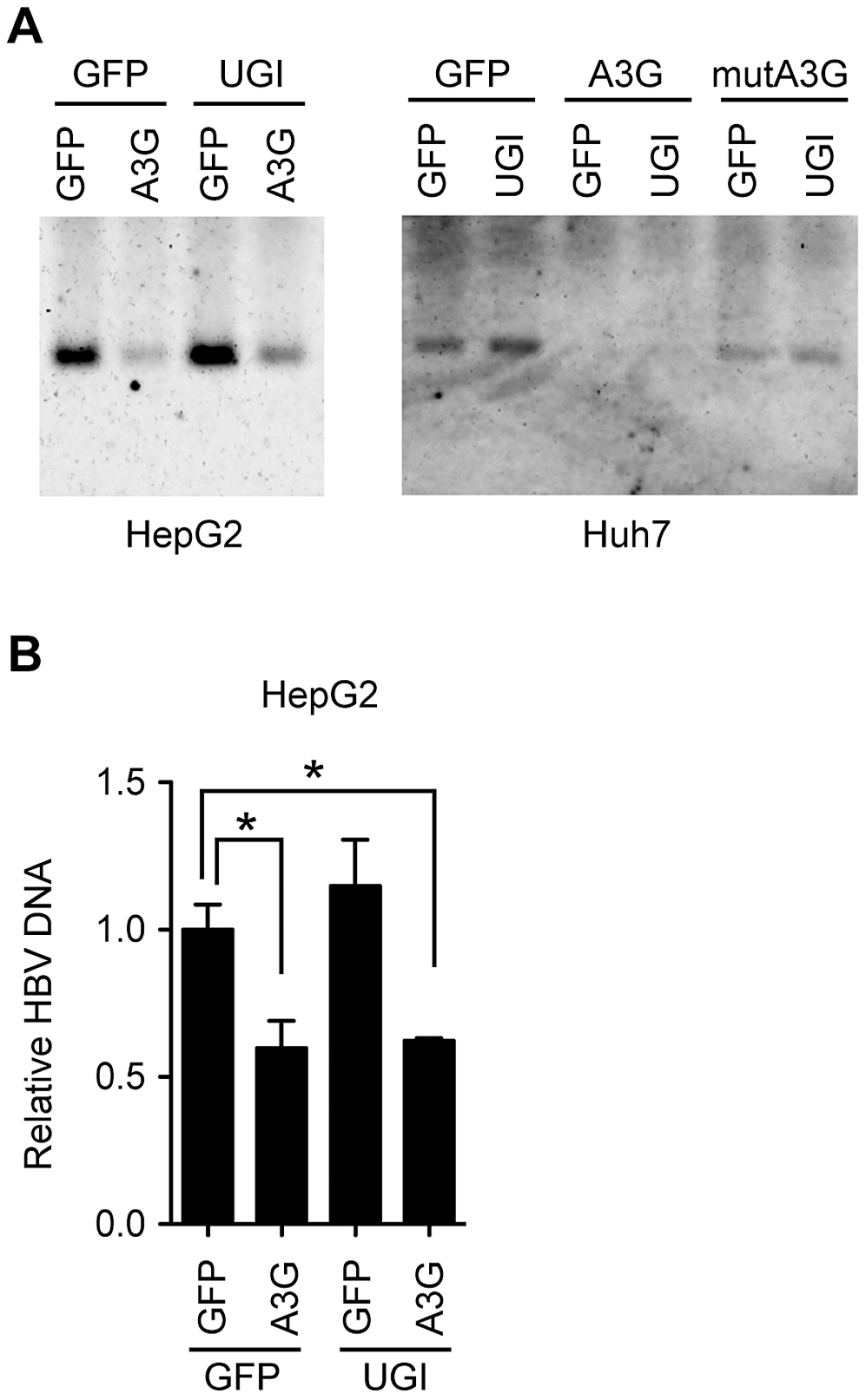
UNG does not affect HBV NC-associated DNA levels during 3 days of culture. (A) Analyses of HBV NCs by NAGE. The CMV-driven HBV plasmid (pPB) and indicated vectors were transfected into human hepatocyte cell lines (Huh7 or HepG2). mutA3G is a deaminase-inactive mutant of A3G (E67Q/E259Q). Cells were harvested 72 h after transfection. Cytoplasmic extracts were subjected to NAGE to measure the HBV NC-DNA by Southern blotting. (B) Quantification of cytoplasmic HBV DNA in HepG2 cells in (A) by qPCR. The level of GFP transfectants was defined as 1-fold. Asterisks indicate statistically significant differences, *P<0.05.

Since UNG inhibition enhanced hypermutation, we next investigated whether UNG inhibition affects another antiviral activity of A3G, the suppression of NC-DNA production. Cytoplasmic HBV NC-DNA was quantified by native agarose gel electrophoresis (NAGE) followed by Southern blotting. NAGE specifically separates intact NC particles, and after the NC particles are transferred to a nylon filter, the DNA content in the NC fraction is measured by hybridization analysis with the HBV DNA probe [21], [22], [48]. As shown in Figure 4A, Southern blotting after NAGE revealed that A3G reduced the NC-DNA content, a result consistent with previous reports [16], [17], [18], [19], [20], [21], [22], [23], [24]. We found that the signal intensity of NC-DNA band from A3G-UGI cotransfectants were slightly higher than that from A3G transfectants in HepG2 cells. However, A3G-mediated reduction was not disturbed by UNG inhibition in Huh7 cells. Quantitative PCR (qPCR) analyses of the NC-DNA revealed no appreciable difference between the presence and absence of UGI in HepG2 cells (Figure 4B). We concluded that UNG did not affect A3G-mediated reduction of NC-DNA, although enhanced hypermutation was observed in the same experimental culture conditions for 3 days.

To observe possible encapsidation of the UNG protein in the HBV NCs, we evaluated the physical association between NC and UNG by immunoprecipitation (IP). Nuclear type of UNG (UNG2) was overexpressed together with the HBV plasmid pPB and FLAG-A3G expression vector in 293T cells. The cytoplasmic fraction containing NCs was used for IP with anti-core (HBc) antibody and Western blotting. As shown in Figure S2A, endogenous mitochondrial UNG (UNG1) was clearly detected in input from the cytoplasmic fractions. Leaked UNG2 from the nucleus was also detected in the input. As reported previously [18], the A3G protein was immunoprecipitated with anti-HBc antibody. However, the signals for UNG2 and GFP disappeared after IP, suggesting that the A3G protein but not the UNG protein is encapsidated into NC. Faint signals for UNG1 were still observed in all lanes of the IP samples at equal signal strength, suggesting nonspecific capture of the UNG1 protein on the protein G sepharose beads. We also investigated the subcellular localization of the UNG2 protein in human hepatocytes. GFP-fused UNG2 localized in the nucleus even in HBV-replicating HepG2 cells (Figure S2B). Taken together, these findings did not reveal evidence for the NC-associated UNG protein, although the NC UNG protein level below detection sensitivity may be sufficient to change the NC-DNA hypermutation frequency.

### DHBV cccDNA was extensively hypermutated by A3G and repaired via the UNG-mediated BER pathway

**Figure 5:**
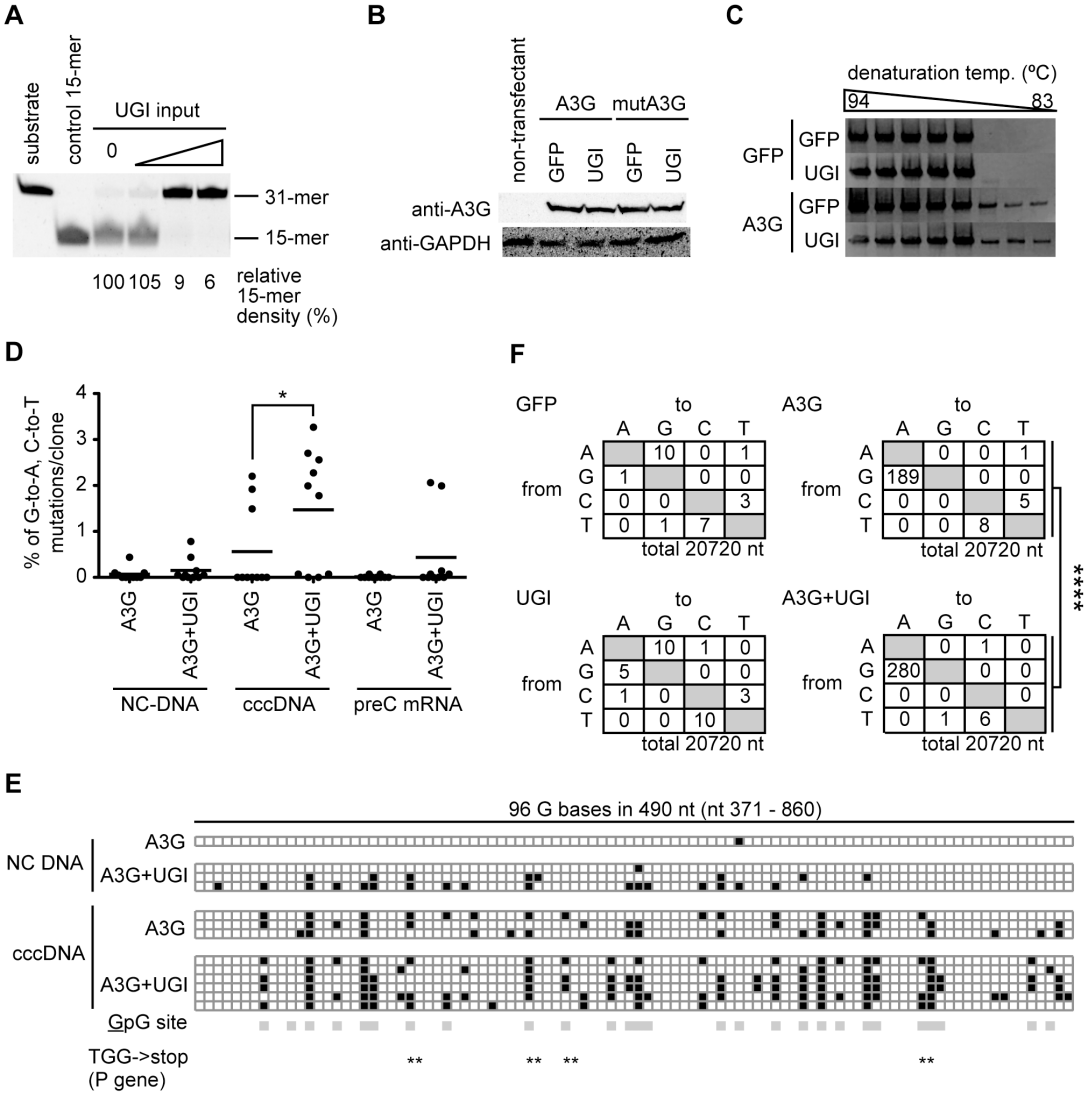
DHBV cccDNA was extensively hypermutated by A3G and repaired via the UNG-mediated BER pathway. (A) Uracil excision activity in the UGI-transfected LMH cells. LMH cells were transfected with different amounts of the UGI vector (1, 2, and 3 µg for 60-mm dish), and after 48-h incubation, uracil excision activities were determined. The signal density for the 15-mer in the lane of the mock transfectant (0 µg of UGI input) was defined as 100%. Synthetic oligonucleotides (substrate and control 15-mer) were also electrophoresed as size markers. (B) Expression levels of exogenous A3G and mutA3G proteins with or without UGI in LMH cells were confirmed by Western blotting with anti-A3G antibody. Expression of GAPDH was also shown to demonstrate equivalent protein loading. (C) The surface-deficient DHBV replicon plasmid (pCSD3.5ΔS) and GFP, A3G, or UGI expression vectors were used to transfect LMH cells as indicated. After 72-h incubation, the cells were harvested and the NC-DNA was purified and amplified by 3D-PCR, using a denaturation temperature gradient of 94–83°C. (D) Frequency of G-to-A and C-to-T mutations in DHBV NC-DNA, cccDNA, and pre-C mRNA. LMH cells were transfected with pCSD3.5ΔS and A3G with or without UGI-expressing vectors. After 6 days of incubation, the cells were harvested. After standard (94°C denaturation) PCR and sequencing, frequencies of G-to-A and C-to-T mutations in NC-DNA (nt 371–2419, the numbering of the nucleotide of the 3021-bp-length DHBV genome starts at the unique EcoRI site), cccDNA (nt 2476–3021/1–860), and cDNA of pre-C mRNA (the same region as cccDNA) were determined and compared. Physical maps are presented in Figure S3A. Each plot represents an independent clone (10 clones for each sample). The average mutation load per clone was indicated by horizontal lines. Asterisks indicate statistically significant differences, *P<0.05. (E) Distribution of G-to-A hypermutation. The boxes in illustration represent 96 G bases within the overlapping sequenced region (490 nt) between NC-DNA and cccDNA. All NC-DNA and cccDNA clones in (D) containing at least 1 G-to-A mutation in the overlapping region are plotted. Black, G-to-A mutation; white, intact G; gray, 5′-GpG-3′ dinucleotide (the underline represents possible mutation target). Position of the TGG codon of the P gene is indicated by double asterisks. The TGG codon can be converted to a premature stop codon when G-to-A conversion occurs. (F) Mutation matrices of the cccDNA. The longer cccDNA fragments (2960 bp; nt 2624–3021/1–2562) were amplified with standard PCR using the same cell source as in (D) and sequenced. DNA sequences that cover almost the complete DHBV genome (3021 bp, full-length) from 7 clones were analyzed for each sample. ****P<0.001. The statistical significance for the frequency of G-to-A mutations was calculated by chi-square test.

We next investigated whether the nuclear viral intermediate, cccDNA, is a target of UNG activity. Since analysis of cccDNA has been difficult using our HBV *in vitro* model because of the low abundance of cccDNA (data not shown), we exploited the DHBV replication system, which efficiently produces cccDNA for analysis [9], [10]. In this study, the surface-deficient DHBV replicon plasmid pCSD3.5ΔS was used to transfect a chicken hepatocyte cell line, LMH, because deficiency of surface protein leads to accumulate more cccDNA than wild-type DHBV [10], [49]. The UNG assay indicated that UGI transfection resulted in efficient decrease in UNG activity even in the LMH cell (Figure 5A). Inhibition of chicken UNG by UGI has been reported previously [50], [51]. Western blotting confirmed that the expression levels of A3G and catalytically inactive mutant (mutA3G) transgenes were not influenced by UGI (Figure 5B). The DHBV plasmid was cotransfected with A3G and UGI vectors into LMH cells. After 3 days, the cells were harvested and NC-DNA was analyzed by 3D-PCR [23] (primer position is indicated in Figure S3A). Data indicate that amplification occurred at the lowest temperature (83°C) from A3G transfectants both with and without UGI expression (Figure 5C), indicating that the A3G protein can hypermutate DHBV NC-DNA. NC-DNA fragments amplified by a standard PCR (94°C) were cloned into the T vector and 10 clones were randomly selected for DNA sequencing. These sequences are shown in Figure 5D (indicated as “NC-DNA”) and Figure S3B. Consistent with other HBV experiments (Figure 3), these data demonstrate the enhanced hypermutation of NC-DNA by UNG inhibition.

Next, cccDNA was isolated by nuclear Hirt extraction and further treated with DpnI to digest transfected plasmids. A cccDNA fragment (1.4 kb) was amplified by standard PCR (94°C) using cccDNA-selective primers spanning the gap region of rcDNA (Figure S3A) [52], [53]. We tested the amplification efficiency of DNAs from nuclear Hirt extract, secreted viral particle, and the replicon plasmid to verify the specificity of cccDNA-selective PCR (Figure S3C). As expected, the 1.4 kb fragment was predominantly amplified from the nuclear Hirt extract containing cccDNA. We also evaluated the specificity of the cccDNA-selective PCR with a replication-defected DHBV replicon plasmid (pCSD3.5ΔP). The cccDNA-selective PCR amplified the 1.4 kb fragment from transfectants of the replication-competent plasmid but not from those of replication-defective plasmid (pCSD3.5ΔP) (Figure S3D). The result clearly demonstrates that our cccDNA-selective PCR amplifies the 1.4 kb from nuclear viral DNA but not from replicon plasmid.

We compared the mutation frequency of cccDNA with those of NC-DNA and pre-C mRNA. Ten randomly selected clones from the A3G-UGI cotransfectants and A3G alone were sequenced, and the mutation frequencies were compared (Figure 5D). Surprisingly, cccDNA clones from the A3G-UGI cotransfectants were hypermutated much more extensively than NC-DNA. Increase in the G-to-A mutation frequency was observed not only in the mutation load per clone but also in the number of clones harboring hypermutation. Eight of 10 cccDNA clones from A3G-UGI cotransfectants carried at least 1 G-to-A/C-to-T mutation, whereas hypermutation was much less frequent on the cccDNA derived from the A3G transfectants. Interestingly, enhanced hypermutations by A3G-UGI were also observed in cDNA clones derived from pre-C mRNA (Figure 5D). The pre-C mRNA is transcribed from cccDNA but not from the replicon plasmid. Overlapping sequences of 2 PCR-amplified regions for NC-DNA and cccDNA (Figure 5E) showed that hypermutation was distributed throughout the P gene and that its distribution patterns were similar between samples. Extensive G-to-A hypermutation by A3G and UGI in this region was confirmed. As expected, mutations were biased toward the GpG dinucleotide, a preferential target of A3G (the underlined nucleotide represents a mutation position) [28]. Considering the dinucleotide preference, the in-frame TGG codon must be susceptible to nonsense mutations (TGA, TAG, and TAA). In this sequence analysis, 6 of 10 clones of cccDNA from A3G-UGI cotransfectants had premature stop codons in the viral P gene open reading frame (ORF), suggesting an effect on downregulation of viral replication. To estimate the overall mutation frequency of the cccDNA, 2.9-kb PCR fragments corresponding to 98% of the whole viral genome were amplified by standard PCR (94°C) and cloned in the T vector. Seven clones were randomly selected from each group and sequenced. Mutation matrices of cccDNA are shown in Figure 5F. As expected from Figure 5D, much more frequent G-to-A mutations were detected in the cccDNA from A3G-UGI cotransfectants than in those from other samples. We observed similar mutation frequencies between the C and P genes (data not shown). Only a few mutations (2 mutations in 7920 nt) in the neomycin-resistant gene of the transfected plasmid (Figure S3E) were detected. The results indicate that cccDNA was extensively hypermutated, while the transfected plasmid was not, in A3G-UGI cotransfectants.

Since nuclear cccDNA was highly mutated by A3G and UGI coexpression, we investigated the subcellular localization of A3G in LMH cells. Microscopic observation revealed that the majority of GFP–A3G fusion proteins were localized in the cytoplasm and that the nuclear A3G protein was not obvious even in DHBV-replicating LMH cells (Figure S4). The data indicate that even the A3G protein in LMH cells localizes in the cytoplasm in a manner similar to that in mammal cells [18], [54], [55], [56] and that the extensive hypermutation in cccDNA is not due to misregulation of A3G intracellular localization.

### UNG inhibition reduced DHBV replication in the presence of A3G

**Figure 6:**
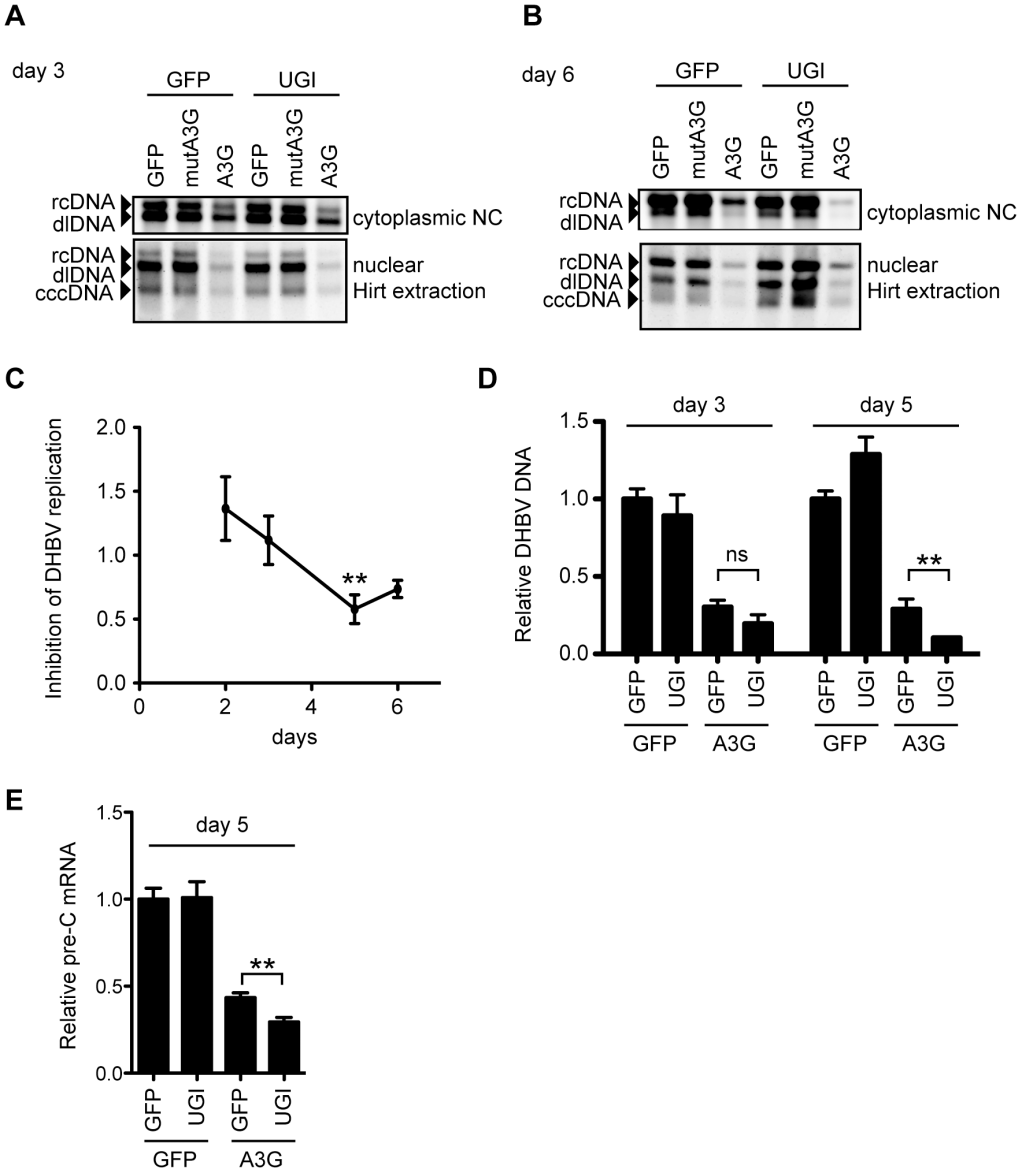
UNG inhibition affects DHBV replication during 6 days of culture. (A and B) Southern blotting for cytoplasmic and nuclear DHBV DNAs at day 3 (A) or day 6 (B) after transfection, with the same conditions as in Figure 5. NC-DNA (rcDNA and double-strand linear [dl]DNA) from the cytoplasmic fraction and Hirt-extracted DNA from the nuclear fraction were purified from the indicated transfectants and subjected to Southern blotting with a DHBV probe. (C) The wild-type DHBV replicon plasmid (pCSD3.5) and A3G vectors were cotransfected with or without the UGI vector. At days 2–6 after transfection, DHBV DNA from secreted viral particles was purified and measured by qPCR assay. Graph shows the relative DHBV DNA level from the A3G-UGI transfectants in the time course. The DNA level from the A3G transfectants was set as 1. **P<0.01. (D) Relative DNA levels of the secreted DHBV. LMH cells were transfected with indicated vectors. DHBV DNAs from secreted viral particles at days 3 and 5 after transfection were purified and measured by qPCR assay. Vertical axis shows the relative DHBV DNA level from the indicated transfectants. The DNA level from the GFP transfectants of each time point was set as 1. **P<0.01. (E) qRT-PCR to determine the expression levels of pre-C mRNA at day 5 in indicated LMH transfectants. The pre-C cDNA level from the GFP transfectants was set as 1. **P<0.01.

To assess the role of UNG in the A3G-induced suppression of DHBV replication, cytoplasmic and nuclear viral DNAs were analyzed by Southern blotting at days 3 and 6 after transfection. A3G-induced suppression was obvious in all samples of NC-DNAs and cccDNAs from both days (Figure 6A and B). The suppression occurred in a deaminase-dependent manner, given that the mutant A3G did not reduce the DHBV DNA levels. Similar to the HBV result in Figure 4, UNG inhibition by UGI did not affect the A3G-mediated reduction of NC-DNA and cccDNA at day 3. However, at day 6, UGI expression enhanced the NC-DNA reduction of A3G-expressing cells, while the cccDNA level of the same transfectants was slightly increased (Figure 6B, lanes 3 and 6). We also performed qPCR to analyze the secreted virion DNA levels from day 2 to 6 after transfection of wild-type DHBV, A3G, and UGI vectors (Figure 6C and D). Culture supernatant was collected daily and viral particles were precipitated by polyethylene glycol (PEG) precipitation. Purified DNA from the precipitants was treated with DpnI to digest any contaminating plasmids. In comparison with A3G alone, secreted viral particle-associated DNA levels in cells cotransfected with A3G and UGI were not significantly different at days 2 and 3. However, consistent with the cytoplasmic Southern blotting data, at day 5, the level of secreted viral DNA in A3G and UGI cotransfectants was lower than that of A3G transfectants (Figure 6C and D).

We also analyzed pre-C mRNA expression levels in day 5 samples. Since pre-C mRNA is transcribed from cccDNA but not from the replicon plasmid, pre-C mRNA expression reflects functional activity of the upstream viral intermediate, cccDNA. As shown in Figure 6E, qRT-PCR showed a consistent reduction with result of Figure 6D.

**Figure 7:**
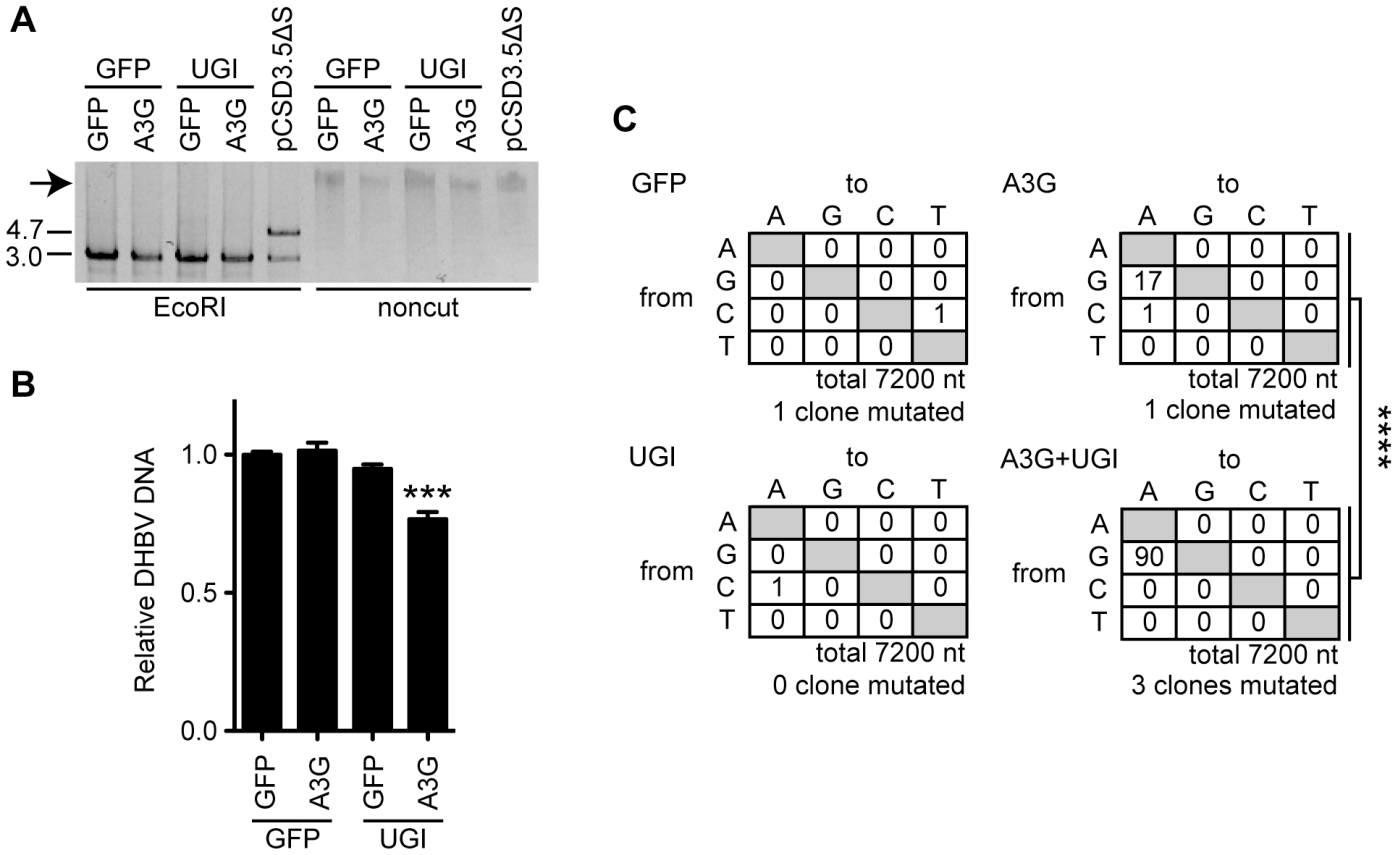
UNG inhibition decreases the replication activity of DHBV cccDNA in the presence of A3G expression. (A) RCA products from the cccDNAs. Expression vectors of A3G, UGI, and GFP were used for transfection of LMH cells together with the pCSD3.5ΔS replicon plasmid. After 7 days of cultivation, cccDNAs were purified from the nuclear fraction by Hirt extraction and treated with DpnI to digest transfected plasmids. The cccDNAs were amplified with phi29 DNA polymerase. The DHBV replicon plasmid (pCSD3.5ΔS) was also reacted as a control. RCA concatemeric products (indicated by an arrow) were digested with EcoRI and electrophoresed on agarose gel to verify successful amplification of the 3.0-kb full-length DHBV genomic DNA (left side). The 4.7-kb fragment represents the pCSD3.5 backbone (see Figure S3A for the plasmid construct). (B) qPCR analysis to assess replication activity of reconstructed replicon plasmids. The amplified full-length genomes from cccDNA were cloned into a pCSD3.5 backbone. Resulting reconstructed clones were used to transfect LMH cells without any other vectors (see Figure S5 for the experimental design). DHBV NC-DNA was purified and quantified 3 days later. The graph shows the relative DHBV DNA level; the level of GFP transfectants was set as 1. ***P<0.005. (C) Mutation matrices for DHBV DNA of reconstructed replicon plasmids. A partial sequence of the P gene (720 bp) from 10 reconstructed replicon plasmid clones was analyzed for each sample. The number of clones containing C-to-T and G-to-A mutations is indicated below. ****P<0.001. The statistical significance for the frequency of G-to-A mutations was calculated by chi-square test.

To evaluate the outcome of cccDNA hypermutation by A3G expression and UNG inhibition, we used rolling circle amplification (RCA), a method widely used to prove the presence of covalently closed circular DNA, including cccDNA of HBV and episomes of human papillomavirus [57], [58] (Figure 7A and S5). cccDNAs were purified from cells 7 days after transfection and then treated with DpnI. The cccDNA was amplified by RCA and digested with EcoRI to cleave the concatemer into individual full-length viral genomes (3.0 kb, Figure 7A). The samples containing cccDNA show 3.0-kb bands (Figure 7A, lanes 1–4), whereas a control RCA reaction of the DHBV plasmid (lane 5) shows both 3.0-kb and 4.7-kb bands. Amplification of the 3.0-kb band without the 4.7-kb DNA (Figure 7A, lanes 1–4) indicates specific amplification of cccDNA but not of the replicon plasmid. The 3.0-kb DNAs were cloned into the replicon plasmid backbone to reconstruct the DHBV plasmids. Reconstructed 20 clones from each sample were pooled and transfected into LMH cells to measure viral replication activity. Importantly, this secondary transfection was performed without A3G and UGI expression vectors. At day 3 after transfection, cytoplasmic NC-DNA was quantified by qPCR (Figure 7B). The reconstructed plasmid with cccDNA from A3G-UGI transfectants showed a significant decrease in NC-DNA production. Consistent with this data, sequence analysis of the P gene in the reconstructed DHBV plasmid revealed that extensive G-to-A hypermutation had accumulated in the RCA products from A3G-UGI cotransfectants (Figure 7C). The data shown in Figure 7B and C may underscore the real impact of hypermutation, given that only 23.8% (720 bp) of the full viral genome per clone was sequenced and that any destructive mutations on the DHBV promoter region would have been rescued by the CMV promoter provided by the backbone in the reconstructed plasmids (Figure S5). Considering all the data, we concluded that nuclear UNG activity repaired uracil bases in cccDNA that were generated by the action of A3s.

## Discussion

To avoid the mutagenic impact of dUTP misincorporation or cytosine deamination, organisms have dUTPase and uracil DNA glycosylases, including UNG. Some viruses such as poxviruses (vaccinia viruses) or herpesviruses (HSV-1 and cytomegaloviruses) also encode UNG homologs, and primate lentiviruses incorporate host UNG into the virion through interaction with the viral Vpr protein [29], [32], [59]. However, the involvement of uracil excision activity during replication and infection of these viruses has not been fully investigated. The effect of Vpr-bound UNG on the deaminated HIV-1 DNA is apparently controversial, although it is thought to be involved in DNA repair [31], [32] or DNA degradation [33] or to have no role [34], [35], [36]. Thus, it seems that there is no unified view whether UNG plays a protective or suppressive role in viral replication. In this study, using *in vitro* models of HBV and DHBV, we investigated the role of UNG activity in hypermutation and viral replication in the presence of A3G. We found that UNG inhibition resulted in the enhancement of A3G-induced NC-DNA hypermutation. This study for the first time also found that the A3G protein induced cccDNA hypermutation (Figures 5 and 7). The cccDNA hypermutation was enhanced on UNG inhibition and subsequently resulted in significant decrease in viral production (Figure 7), suggesting a protective role of UNG for viral replication against cccDNA hypermutation.

It has been difficult to determine which HBV intermediates are catalyzed by UNG because HBV shuttles between the cytoplasm and the nucleus during its life cycle. Plasmids can also act as a target molecule. Stenglein *et al.* showed that APOBEC3A induces C-to-U hypermutation in transfected plasmids and that uracilated plasmids were processed by UNG activity using UGI-expressing 293T cells [60]. In the present study, APOBEC3A was not expressed in HepG2 and LMH cells (Figure 2B). The contaminated DHBV plasmid does not contribute to the results of cccDNA sequencing (Figures 7A and S3C–E). Comparison of hypermutation frequencies of NC-DNA, cccDNA, and pre-C mRNA from the same cell source revealed that the mutation frequency of cccDNA was higher than that of NC-DNA. G-to-A mutations were predominant in cccDNA clones (Figure 5), whereas C-to-T mutation is a characteristic feature of plasmid hypermutation [60]. The transfected vector backbone did not accumulate hypermutation (Figure S3E). Accordingly, we concluded that A3G does not target the replicon plasmid and that the mechanism of cccDNA hypermutation differs from that of plasmid hypermutation [60]. The observed G-to-A hypermutation is one of the prominent features of cccDNA hypermutation. Predominant G-to-A hypermutation in NC-DNA (Figures 5 and S3B), a precursor of cccDNA, may partly account for the G-to-A cccDNA hypermutation. However, it does not explain the higher mutation frequency of exclusive G-to-A hypermutation in cccDNA than that in NC-DNA. Further study is required to clarify the mechanism of cccDNA hypermutation.

A3G is known to be encapsidated into NC and deaminate NC-DNA. We propose that an additional deamination event by A3G may occur during or after cccDNA formation in the nucleus. Although the nuclear localization of GFP-A3G was not obvious in DHBV-replicating LMH cells (Figure S4), the encapsidated A3G may be able to enter the nucleus in the same manner as viral rcDNA. Indeed, a recent HIV-1 study showed that infection with Vif-deficient HIV-1 led to uracil accumulation in the host genome, implying that Vif-sensitive A3s, including A3G, can also deaminate nuclear DNA [61]. We found that A3G and UGI coexpression caused extensive nuclear cccDNA hypermutation (Figures 5 and 7). Neither a UNG nor a Vpr homolog has been identified in HBV, unlike the HIV-1 genome, and we failed to detect UNG proteins in HBV NC (Figure S2). It is reasonable to suggest that UNG excises uracils in the nucleus, resulting in extensive nuclear cccDNA hypermutation in A3G-UGI transfectants.

A proposed antiviral role for UNG is DNA degradation following DNA cleavage of an abasic site generated by uracil excision [13], although UNG is primarily considered a DNA repair factor. In the present study, we compared viral production between UGI presence and absence in A3G-expressing hepatocytes. Those experiments did not show obvious rescue of A3G-mediated suppression of viral replication by UNG inhibition (Figures 4 and 6). The A3G-mediated reduction of NC-DNA was enhanced by UNG inhibition in prolonged culture (Figure 6B–E), coincidentally with extensive hypermutation of cccDNA (Figure 5). In this experimental setting, UNG-dependent DNA degradation was not obvious. We accordingly propose that UNG removes uracil residues on/after cccDNA formation in the nucleus and that these uracil excisions contribute to reducing dysfunctional mutagenesis induced by APOBEC deaminases (Figure S6). Observation of hypermutated pre-C mRNA (Figure 5D) suggests that hypermutated pgRNA is also transcribed from the cccDNA and may contribute to the enhanced hypermutation in NC-DNA by UNG inhibition (Figures 3 and S3B).

Previous *in vivo* studies showed no evidence of cytidine deamination in the DHBV genome during chronic infection [62]; however, cccDNA sequences were not analyzed. In the present study, several G-to-A/C-to-T mutations in DHBV cccDNA were observed in LMH cells even without A3G overexpression (Figure 5F), suggesting the contribution of endogenous deaminase activity. The chicken genome possesses only 3 AID/APOBEC members: AID, APOBEC2 (A2), and APOBEC4 (A4) [13], [56], [63]. In mammals, A2 is considered to play a role in muscle development, and A4 may not have deaminase activity [54], [56]. There is a functional association of AID and UNG with immunoglobulin gene diversification in human, mouse, and chicken B cells [12], [29], [30], [51]. Recent reports showed that human AID endogenous expression was detected in hepatocytes after TGF-β stimulation [64], and overexpression of AID caused HBV hypermutation of NC-DNA [24]. In future studies, we plan to assess the contribution of AID to antiviral activity against HBV and DHBV.

APOBEC3G-mediated hypermutation of Vif-deficient HIV-1 caused deleterious effects on viral replication, whereas partial Vif activity accelerated viral diversification [65], [66]. Similarly, we speculate that the balance between AID/APOBECs and UNG activities on mutation frequency decides the consequence to hepadnaviruses: deleterious mutations vs. diversification. DNA sequencing showed the existence of several cccDNA clones having a single G-to-A or C-to-T mutation (Figure 5D), a finding that favors the concept of generation of clonal diversity by APOBEC and UNG proteins. This study suggests a possible role of APOBEC proteins as a mutator of HBV cccDNA. Because mutation in cccDNA is direct resource of viral variants, we are currently exploring the possibility that APOBECs and BER factors are involved in the emergence of drug-resistant mutants of HBV and DHBV.

## Materials and Methods

### Plasmids

The HBV replicon plasmids pHBV1.5 and pPB express all the HBV viral gene products necessary for viral replication and are under the control of the HBV and CMV promoters, respectively [40], [41], [44]. The DHBV replicon plasmid pCSD3.5 was generated by insertion of DHBV viral genomic DNA (equivalent to 1.17-mer) into pCMV-script (Stratagene) (kindly provided by Dr. K. Kuroki, Kanazawa University) [49]. The pCSD3.5ΔS plasmid was generated by introduction of in-frame stop codons at positions 1327, 1346, and 1349 in the surface protein ORF without affecting the P gene ORF [49]. Other expression vectors are listed in Table S1.

### Cell culture, treatment, retrovirus-mediated gene transduction, and transfection

Cells (HepG2, Huh7, 293T, and LMH) and subsequent transfectants were grown and maintained in Dulbecco's modified Eagle medium (DMEM; Sigma) containing 10% fetal bovine serum, 100 U/mL penicillin, and 100 µg/mL streptomycin. IFNγ (recombinant IFNγ-1a) was purchased from Shionogi. Tamoxifen (Wako) was dissolved in ethanol (EtOH). A stable line of HBV-expressing HepG2 cells was established using a standard method [11]. In brief, HepG2 cells were transfected with linearized pPB, and G418-drug selection with limiting dilution was performed. Of the resulting transfectants, a cell line with a high level of HBV NC-DNA was chosen among G418-resistant clones. Retrovirus-mediated gene transduction was performed as described previously [38], [67]. In brief, retroviral vectors were transfected into packaging platA cells (Cell Biolabs), and virus-containing culture supernatants were used for infection of HepG2 cells. Two days after infection, 1 µg/mL puromycin (Wako) was added to eliminate uninfected cells. Plasmid transfection was performed using Fugene 6 (Roche) according to the manufacturers' instructions. The total DNA amount (6 µg for 60-mm dish) for each transfection was kept constant by adding GFP vector.

### UNG assay

The UNG assay was performed as described previously, with minor modification [39]. Cells were resuspended in HE buffer [25 mM Hepes-KOH (pH 7.8), 1 mM EDTA, 1 mM DTT, 10% glycerol] and fractured by freezing in liquid nitrogen and thawing. A fluorescein isothiocyanate (FITC)-labeled 31-mer oligonucleotide containing a central dU residue (5′-AGCTTGGCTGCAGGTUGACGGATCCCCGGGA-3′) was synthesized as a substrate. An FITC-labeled 15-mer oligonucleotide (5′-AGCTTGGCTGCAGGT-3′) was also synthesized for use as a molecular size marker. Approximately 10 pmol of substrate was incubated with cell extracts for 2 h, and the resulting abasic sites were cleaved with alkali and heat treatment. The reaction products were separated by 6 M urea/20% polyacrylamide gel electrophoresis. An FITC signal was visualized in an LAS imager system (FujiFilm) and quantified by densitometry using ImageJ software.

### Purification of HBV and DHBV DNA

Cytoplasmic HBV NC-DNA was purified as reported by Gunther *et al.*, with minor modifications [68]. In brief, the cells were lysed with buffer [10 mM Tris-HCl (pH 8.0), 1 mM EDTA, 1% NP-40, 8% sucrose, proteinase inhibitor cocktail (Roche)]. After centrifugation, cytoplasmic supernatants were collected and further treated with DNase I and RNase A. NCs were PEG-precipitated and digested with proteinase K and sodium dodecyl sulfate (SDS). Secreted DHBV particles were also precipitated with PEG8000, followed by DNase I treatment and digestion with proteinase K and SDS to extract viral DNA. For purification of DHBV DNAs, LMH cells were lysed in 0.5% NP40 lysis buffer, and the nuclei were collected by centrifugation to separate cytoplasmic and nuclear fractions. The cccDNA extraction from the nuclear fraction was performed using a modified Hirt extraction procedure [69]. The nuclear pellet was lysed in 50 mM Tris–HCl (pH 7.5), 10 mM EDTA, and 2% SDS. After 20 min incubation at room temperature, 0.5 M KCl was added to the lysate and incubated at 4°C overnight. From the supernatant after centrifugation, DNA was purified by phenol∶chloroform extraction and ethanol precipitation. All purified DNA solutions were treated with DpnI restriction enzyme to digest any contaminating plasmid DNA.

### Hypermutation analysis

The 3D-PCR procedure was performed as described previously, with minor modifications [18], [28]. Primers used for 3D-PCR are shown in Table S2. For 3D-PCR of HBV, the first PCR was performed as follows: 94°C for 5 min, 35 cycles of 94°C for 30 s, 50°C for 30 s, and 72°C for 30 s, and a final elongation step at 72°C for 3 min. The nested PCR was performed as follows: 94–83°C for 5 min, 35 cycles of 94–83°C for 60 s, 45°C for 30 s, and 72°C for 30 s, and a final elongation step of 72°C for 3 min. For DHBV NC-DNA, 1 round of 3D-PCR was performed using primers indicated in Figure S3A [23]. Initial denaturation was for 5 min at 94–83°C, followed by 35 cycles of 30 s at 94–83°C, 30 s at 55°C, and 2 min at 72°C, with a final elongation for 7 min at 72°C. For standard (94°C) PCR of cccDNA, a cccDNA-selective primer set was used (Figure S3A and Table S2) [52], [53]. Specific amplification of cccDNA is shown in Figures S3C and S3D. To determine the hypermutation frequency, PCR fragments from 3D-PCR or standard PCR were cloned into T vectors (Promega), and the indicated number of successful recombinant clones was randomly selected and sequenced using ABI PRISM 3130 (Applied Biosystems).

### Quantification of expression levels of transcripts and viral DNAs

Total RNA was extracted using TRIsure (Bioline), treated by amplification grade DNase I (Invitrogen), and reverse-transcribed using an oligo-dT primer and the SuperScript III kit (Invitrogen). For DHBV pre-C mRNA, a DHBV-specific primer was used in the reverse transcription reaction. qPCR analysis was performed using SYBR Premix Ex Taq (Takara) on an MX3000 thermocycler (Stratagene) following the PCR protocol. Human AICDA, A3B, A3C, A3DE, A3F, A3G, A3H, HPRT, DHBV pre-C, and chicken HPRT expression levels were measured using PCR conditions of 95°C for 1 min; 40 cycles of 95°C for 15 s, 55°C for 30 s, and 70°C for 30 s; and 1 cycle of 95°C for 1 min, 55°C for 30 s, and 95°C for 30 s. For A3A amplification, an annealing temperature of 60°C was used.

For analysis of purified viral DNAs, qPCR was performed using the following conditions: HBV, 40 cycles of 95°C for 15 s, 52°C for 30 s, and 70°C for 30 s; DHBV, 40 cycles of 95°C for 5 s and 60°C for 20 s. HBV and DHBV DNA copy numbers were determined using a pPB or pCSD3.5 plasmid standard curve, respectively. Amplified fragments were designed to contain at least 2 DpnI sites to avoid amplification from contaminated plasmids. Primer sequences are listed in Table S2.

### Western blotting

Cells were lysed in SDS sample buffer, sonicated, boiled, separated by 12% SDS-PAGE, and then transferred to a Hybond ECL membrane (Amersham). The membrane was incubated in a blocking buffer of 5% skim milk in phosphate-buffered saline containing 0.1% Tween 20. Signals were detected using the LAS1000 imager system. The antibodies used in this study were as follows: rabbit anti-A3G (Sigma; raised against A3G peptide [CQDLSGRLRAILQNQEN]), rabbit anti-GAPDH (G9545, Sigma), mouse anti-FLAG (M2, Sigma), rabbit anti-ER (HC-20, Santa Cruz Biotechnology), rabbit anti-UNG (ab23926, Abcam), rabbit anti-HBc (B0586, Dako), anti-rabbit Igs-horseradish peroxidase (HRP) (ALI3404, eBiosource), and rabbit and mouse IgG Trueblot (eBioscience).

### NAGE and Southern blotting for viral DNAs

NAGE analysis was performed as described previously [21], [22], [48]. In brief, crude cytoplasmic extracts containing HBV NC particles were loaded into a 1% agarose gel for electrophoresis to separate intact capsid particles. After electrophoresis, the NC particles were denatured with NaOH and transferred onto a nylon membrane. HBV and DHBV DNAs were detected using a double-stranded HBV and DHBV DNA probes spanning the entire viral genome, respectively. Probe labeling and signal development were performed using the AlkPhos direct labeling system (Amersham), and the signals were detected using the LAS1000 imager system.

### RCA of DHBV cccDNA

Margeridon *et al.* previously demonstrated that the RCA method specifically amplifies cccDNA with high fidelity but does not amplify any other intermediate DNAs [57]. We followed their method with minor modifications. In brief, DHBV cccDNA purified by Hirt extraction (DpnI treated) was mixed with 8 DHBV-specific primers (Table S2); denatured at 95°C for 3 min; cooled sequentially at 50°C for 15 s, 37°C for 15 s, and room temperature; and reacted with the phi29 DNA polymerase and buffers (New England Biolabs) at 37°C for 16 h. Note that the DNA polymerase (phi-29 DNA pol) used in the RCA reaction can polymerize DNA even with a uracil-containing DNA template [70]. The RCA concatemerized product was converted to the monomeric full-length DHBV genome by digestion with EcoRI, where the DHBV sequence contains a single site, and was cloned into the replicon vector backbone at the EcoRI site; thus, the full-length DHBV genome in the original vector was replaced with the corresponding fragment from the purified cccDNA. These reconstructed plasmids were cloned, sequenced, and transfected into LMH cells in order to analyze their replication activities (see Figure S5 for experimental design).

### RNAi analyses

Two UNG-specific siRNAs, two A3G-specific siRNAs and control siRNA (Stealth Select grade) were purchased from Invitrogen and were used to transfect using Lipofectamine 2000, according to the manufacturer's instructions. Cells and viruses were analyzed 48 h after transfection.

### Coimmunoprecipitation

Cells were lysed with IP lysis buffer [50 mM Tris-HCl (pH 7.1), 20 mM NaCl, 1% NP-40, 1 mM EDTA, 2% glycerol, a proteinase inhibitor cocktail (Roche)]. After centrifugation, the supernatants (cytoplasmic fraction) were incubated with anti-HBc antibody (DAKO) and protein G sepharose (Amersham), and passed through a micro BioSpin chromatography column (BioRad). After the column was washed with the lysis buffer, the coprecipitated proteins were used for Western blotting.

### Statistical analysis

Statistical analyses were performed using GraphPad Prism (GraphPad Software). ANOVA analysis was used for qPCR data. The Kruskal–Wallis test with Dunn's post test or Pearson's chi-square test were used for mutation analyses. P values less than 0.05 between experimental groups were considered statistically significant. For all graphs in this study, error bars indicate standard error of the mean from triplicate samples.

## Supporting Information

Figure S1 **siRNA experiments of UNG and A3G.** (A) Uracil excision activity in the siRNA-transfected 293T cells. The 293T cells were transfected with the indicated siRNAs, and after 48-h incubation, uracil excision activities were determined. The signal density for the 15-mer in the lane of 20 nM control siRNA was defined as 100%. (B) Knockdown of UNG expression also enhances hypermutation of HBV NC-DNA. The CMV-driven HBV replicon plasmid (pPB), A3G vector, and the 20 nM siRNAs were transfected into 293T cells. After 48 h, the cells were harvested and the HBV NC-DNA was subjected to 3D-PCR analysis. (C) Quantification of APOBEC3G expression in the IFNγ-stimulated cells. HepG2 cells were transfected with the indicated A3G (or control) siRNAs and after 16 h, cells were stimulated with 1000 U/mL IFNγ for an additional 48 h. qRT-PCR was performed to determine the expression level for A3G. The expression level of control siRNA was defined as a 1-fold change. (D) Alignment of hypermutated HBV sequences. PCR fragments from the 87.2°C denaturation temperature reaction in Figure 2D were excised from agarose gel and cloned into T vectors, and subsequently four random selected clones were sequenced from each sample. The reference sequence from the pPB is shown above. Dots in the alignment represent identity with the reference sequence.
(TIF)

Figure S2 **Immunoprecipitation of NC and nuclear localization of the UNG protein.** (A). To detect any potential physical binding between UNG and core proteins, immunoprecipitation was performed. pPB, FLAG-A3G (or FLAG-GFP), and UNG2 expression vectors were transfected into 293T cells, as indicated. At 48 h after transfection, the cells were harvested and subjected to IP with anti-HBc antibody using cytoplasmic lysates. The crude cytoplasmic extract was also blotted to verify UNG1, UNG2 FLAG-A3G, FLAG-GFP, and core proteins. Nonspecific binding of FLAG-A3G to protein G Sepharose beads was observed (lane 6), but a much stronger signal was observed in lane 8 than in lane 6. Signals for the core protein in the lanes 7 and 8 verified successful immunoprecipitation of the core protein to the IP fraction. Although UNG2 was overexpressed, it was not precipitated by the anti-HBc antibody. (B) Intracellular localization of the UNG protein in HepG2 cells. pEGFP-UNG2 or control pEGFP vector was transfected into HBV stably expressing HepG2 cells. The nucleus was visualized with simultaneous expression of the DsRed-NLS protein that mainly localized in the nucleus. The EGFP protein was distributed in the nucleus and cytoplasm, whereas UNG2 was localized only in the nucleus.
(TIF)

Figure S3 **PCR amplification of DHBV DNAs.** (A) Primer positions to amplify DHBV NC-DNA, cccDNA, pre-C cDNA and the replicon plasmid pCSD3.5 are shown. The viral genome in the NC is a rcDNA form with gaps in both strands (left). (−) and (+) represent minus- and plus-strand DNAs. Dotted line represents region where plus-strand DNA may potentially not be synthesized. In nucleus, the genome is converted into a cccDNA form (right). Primers of pol-f and pol-r amplify both DNA forms. 3D-PCR of NC-DNA in Figure 5C was performed with pol-f and pol-r primers. ccc-f and ccc-r are cccDNA-selective primers that span the gap region of rcDNA. DHBV genes are represented as gray boxes. Primers to detect the cDNA of pre-C mRNA are same as cccDNA-selective primers. Pre-C mRNA is not transcribed from this plasmid but from cccDNA. Primers of neo-f and neo-r amplify the partial sequence of the neomycin-resistant gene of the replicon plasmid. Numbers indicate nucleotide positions of the 3021-bp-length DHBV genome starting at the unique *Eco*RI site. (B) Mutation matrices of the NC-DNA for Figure 5D. The DHBV NC-DNA fragments amplified with the standard PCR (94°C) using pol-f and pol-r were cloned into a T vector. DNA sequences from 10 clones were analyzed for each sample. Pie charts represent the proportion of clones with G-to-A and C-to-T mutations for left-side matrices. The total number of independent clones is indicated in the center. The number of mutations is indicated on the periphery of the pie segment. ***P<0.005. The statistical significance for the frequency of G-to-A mutations was calculated by chi-square test. (C) Selective PCR amplification for cccDNA. The template DNA samples were serially diluted 1/5 and 1/25 and amplified by pol-f/pol-r or ccc-f/ccc-r primer set. P: pCSD3.5 DHBV replicon plasmid starting at 10^10^ copies per reaction. N: nuclear Hirt-extracted DNA from pCSD3.5 transfectant. S: NC-DNA from culture supernatant of pCSD3.5 transfectants. The nuclear Hirt-extracted DNA containing cccDNA shows efficient amplification in both PCR reactions, whereas plasmid and NC-DNAs do not show efficient amplification in cccDNA-selective PCR. (D) The cccDNA-selective PCR for the transfectants of replication-defective replicon plasmid. pCSD3.5ΔS or the replication-defective DHBV replicon plasmid (pCSD3.5ΔP) was transfected into LMH cells. After 3 days incubation, nuclear Hirt extract was prepared by the same procedure as in Figure 5. DNA samples were subjected to the cccDNA-selective PCR. (E) Mutation matrix of the neomycin-resistant gene of the replicon plasmid. pCSD3.5ΔP, A3G and UGI expression vectors were transfected into LMH cells. The Hirt-extracted DNA from transfectants at day 6 was subjected to neo-f/neo-r PCR. PCR products were cloned into a T vector. DNA sequences from 12 clones were analyzed.
(TIF)

Figure S4 **Intracellular localization of the A3G protein in LMH cells.** Surface-deficient DHBV and EGFP-A3G or control EGFP vectors were used to transfect LMH cells. The nucleus was visualized with simultaneous expression of the DsRed-NLS protein. The EGFP protein was distributed in the nucleus and cytoplasm, whereas majority of GFP signals from the EGFP–A3G fusion protein come from cytoplasm.
(TIF)

Figure S5 **Experimental scheme for** **Figure 7** **.** The cccDNAs were purified from the cells 7 days after transfection and then treated with DpnI to digest any contaminating plasmids. The cccDNA was amplified by RCA and digested using EcoRI to produce 1 full-length copy of viral genomic DNA (Figure 7A). These EcoRI fragments were cloned into the replicon plasmid backbone (using the CMV promoter) to reconstruct the DHBV replicon plasmids. After transformation of reconstructed plasmids, 20 transformed and reconstructed *E. coli* clones were selected randomly from each sample. Twenty minipreps for each sample were prepared and DNA concentrations were estimated. From the 20 reconstructed replicon plasmids, 0.5 µg were taken, pooled, and used to transfect LMH cells without A3G or UGI vectors. Three days after transfection, NC-DNA was purified and quantified by qPCR (Figure 7B). For sequence analysis of reconstructed clones, 10 clones were randomly selected from the 20 reconstructed clones and result is shown in Figure 7C.
(TIF)

Figure S6 **A proposed model to explain how UNG reduces uracil load on cccDNA.** Intracellular viral lifecycle together with possible role of UNG. pgRNA is transcribed from cccDNA and the replicon plasmid when transfected. NC is assembled in the cytoplasm from core and P proteins together with pgRNA. In human hepatocytes, interferon induces APOBEC proteins such as A3G. A3G is encapsidated in a subset of NCs and induces hypermutation predominantly on the minus strand of rcDNA, resulting in G-to-A hypermutation. In addition, A3G inhibits minus strand DNA synthesis. After transportation into nucleus, additional hypermutation may be induced by A3G, and UNG repairs them during or after cccDNA formation. When UNG activity is inhibited by UGI, the extensive hypermutation remains in cccDNA, disrupting the genetic information for viral replication. Pre-C mRNA is transcribed from cccDNA but not from the replicon plasmid. When hypermutation does not affect any processes required for transcription, hypermutated transcripts such as pgRNA and pre-C mRNA are transcribed from hypermutated cccDNA. The hypermutated pgRNA may be encapsidated to enter a second viral lifecycle.
(TIF)

Table S1 **List of plasmids used in this study.**
(PDF)

Table S2 **List of primers used in this study.**
(PDF)
