# Supplementary figures and images for: Uracil DNA Glycosylase Counteracts APOBEC3G-Induced Hypermutation of Hepatitis B Viral Genomes: Excision Repair of Covalently Closed Circular DNA
Source: PLoS Pathog. 2013 May 16;9(5):e1003361. doi: 10.1371/journal.ppat.1003361 (PMC3656096; doi:10.1371/journal.ppat.1003361)

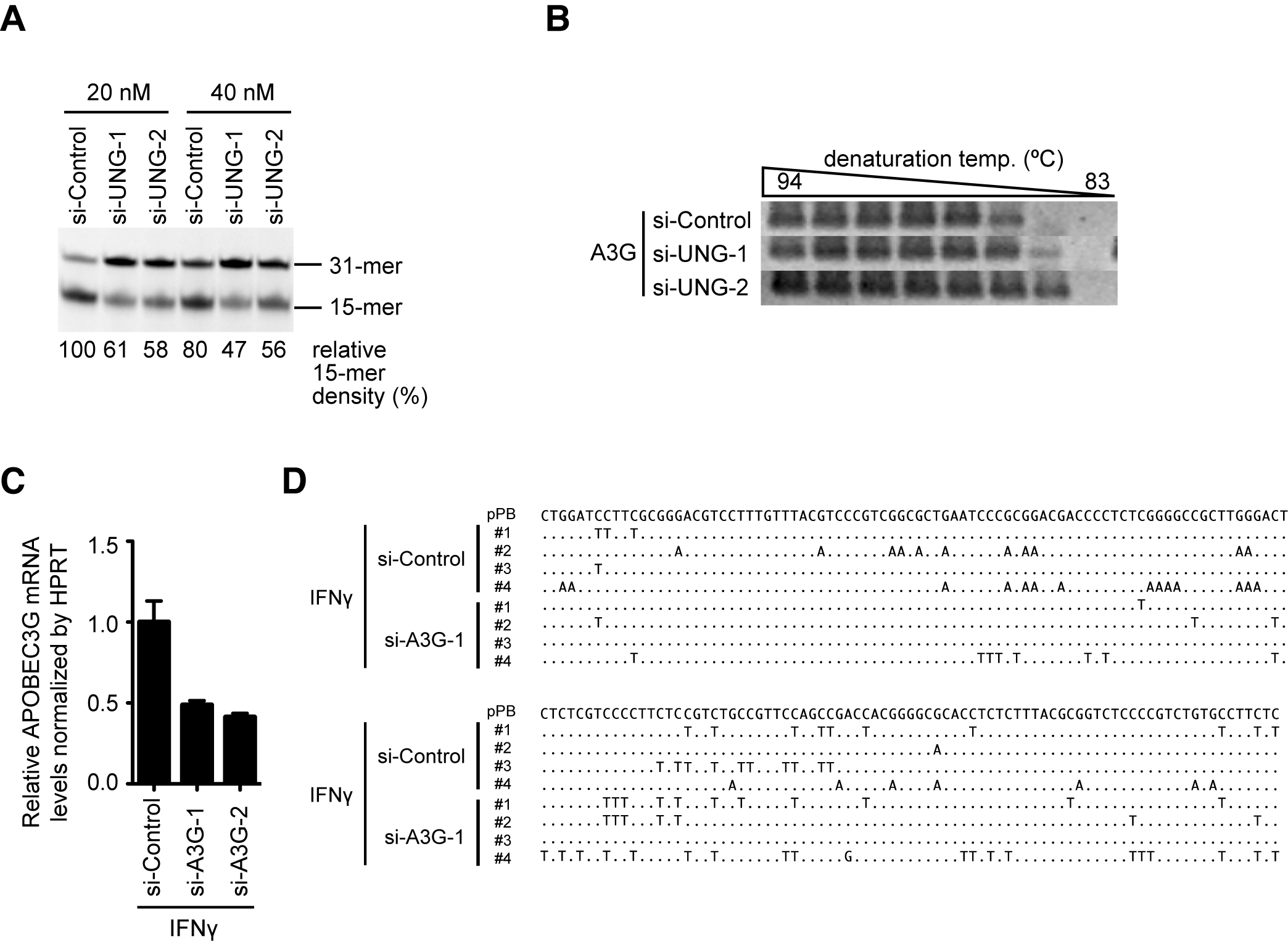

Supplement: Figure S1 — siRNA experiments of UNG and A3G. (A) Uracil excision activity in the siRNA-transfected 293T cells. The 293T cells were transfected with the indicated siRNAs, and after 48-h incubation, uracil excision activities were determined. The signal density for the 15-mer in the lane of 20 nM control siRNA was defined as 100%. (B) Knockdown of UNG expression also enhances hypermutation of HBV NC-DNA. The CMV-driven HBV replicon plasmid (pPB), A3G vector, and the 20 nM siRNAs were transfected into 293T cells. After 48 h, the cells were harvested and the HBV NC-DNA was subjected to 3D-PCR analysis. (C) Quantification of APOBEC3G expression in the IFNγ-stimulated cells. HepG2 cells were transfected with the indicated A3G (or control) siRNAs and after 16 h, cells were stimulated with 1000 U/mL IFNγ for an additional 48 h. qRT-PCR was performed to determine the expression level for A3G. The expression level of control siRNA was defined as a 1-fold change. (D) Alignment of hypermutated HBV sequences. PCR fragments from the 87.2°C denaturation temperature reaction in Figure 2D were excised from agarose gel and cloned into T vectors, and subsequently four random selected clones were sequenced from each sample. The reference sequence from the pPB is shown above. Dots in the alignment represent identity with the reference sequence. (TIF) [file ppat.1003361.s001.tif]

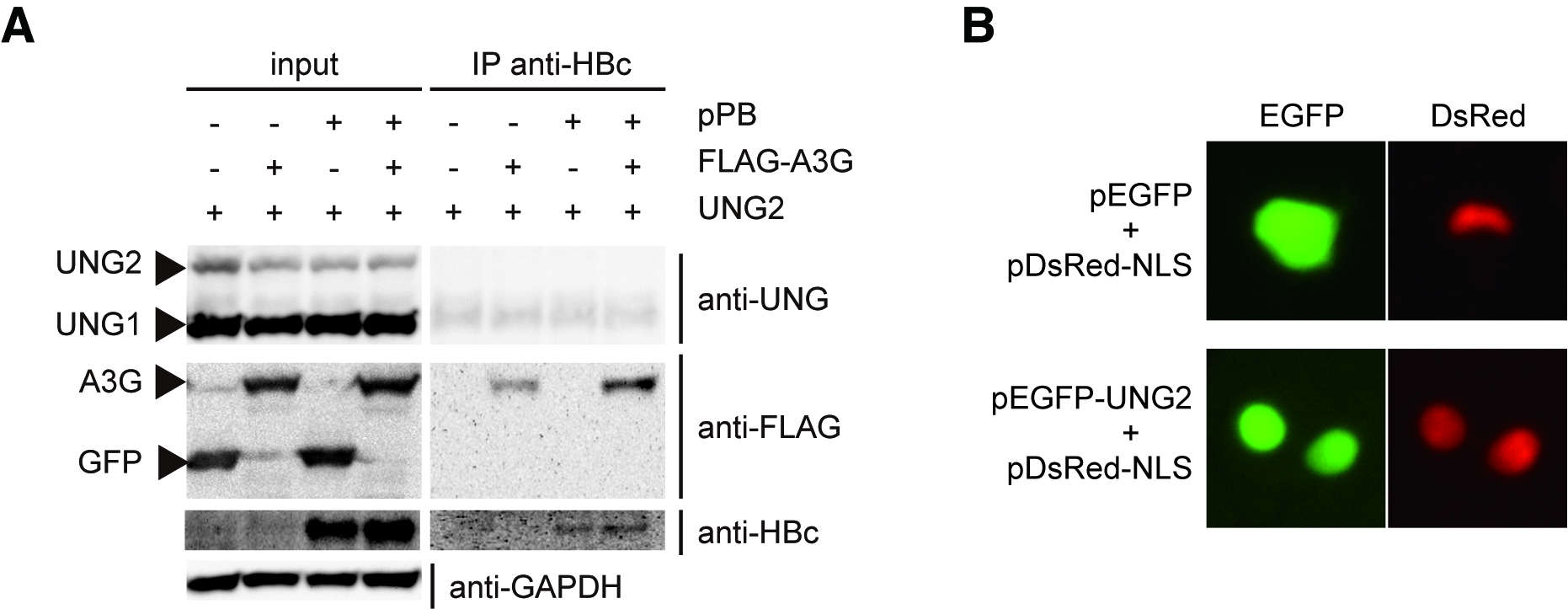

Supplement: Figure S2 — Immunoprecipitation of NC and nuclear localization of the UNG protein. (A). To detect any potential physical binding between UNG and core proteins, immunoprecipitation was performed. pPB, FLAG-A3G (or FLAG-GFP), and UNG2 expression vectors were transfected into 293T cells, as indicated. At 48 h after transfection, the cells were harvested and subjected to IP with anti-HBc antibody using cytoplasmic lysates. The crude cytoplasmic extract was also blotted to verify UNG1, UNG2 FLAG-A3G, FLAG-GFP, and core proteins. Nonspecific binding of FLAG-A3G to protein G Sepharose beads was observed (lane 6), but a much stronger signal was observed in lane 8 than in lane 6. Signals for the core protein in the lanes 7 and 8 verified successful immunoprecipitation of the core protein to the IP fraction. Although UNG2 was overexpressed, it was not precipitated by the anti-HBc antibody. (B) Intracellular localization of the UNG protein in HepG2 cells. pEGFP-UNG2 or control pEGFP vector was transfected into HBV stably expressing HepG2 cells. The nucleus was visualized with simultaneous expression of the DsRed-NLS protein that mainly localized in the nucleus. The EGFP protein was distributed in the nucleus and cytoplasm, whereas UNG2 was localized only in the nucleus. (TIF) [file ppat.1003361.s002.tif]

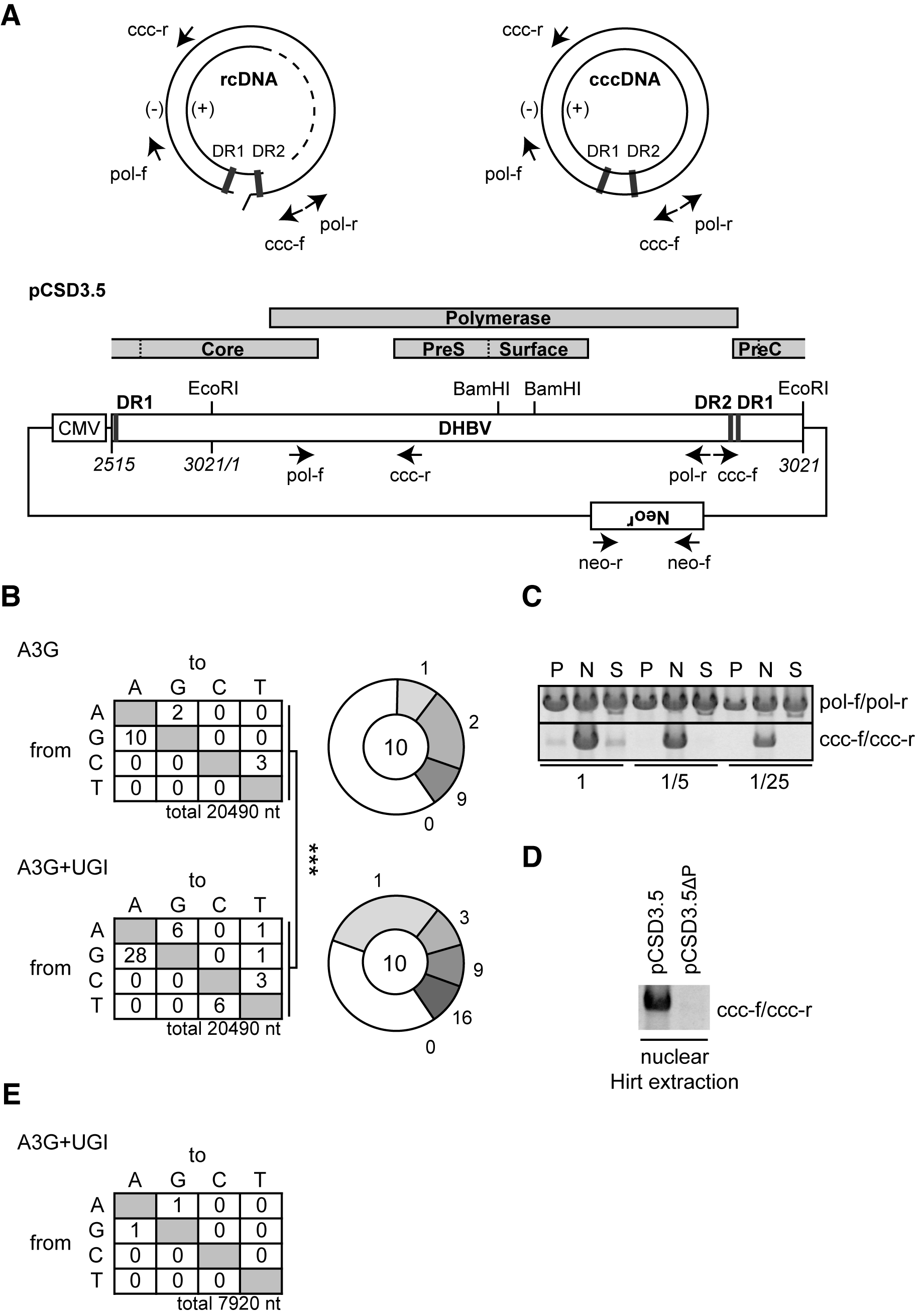

Supplement: Figure S3 — PCR amplification of DHBV DNAs. (A) Primer positions to amplify DHBV NC-DNA, cccDNA, pre-C cDNA and the replicon plasmid pCSD3.5 are shown. The viral genome in the NC is a rcDNA form with gaps in both strands (left). (−) and (+) represent minus- and plus-strand DNAs. Dotted line represents region where plus-strand DNA may potentially not be synthesized. In nucleus, the genome is converted into a cccDNA form (right). Primers of pol-f and pol-r amplify both DNA forms. 3D-PCR of NC-DNA in Figure 5C was performed with pol-f and pol-r primers. ccc-f and ccc-r are cccDNA-selective primers that span the gap region of rcDNA. DHBV genes are represented as gray boxes. Primers to detect the cDNA of pre-C mRNA are same as cccDNA-selective primers. Pre-C mRNA is not transcribed from this plasmid but from cccDNA. Primers of neo-f and neo-r amplify the partial sequence of the neomycin-resistant gene of the replicon plasmid. Numbers indicate nucleotide positions of the 3021-bp-length DHBV genome starting at the unique EcoRI site. (B) Mutation matrices of the NC-DNA for Figure 5D. The DHBV NC-DNA fragments amplified with the standard PCR (94°C) using pol-f and pol-r were cloned into a T vector. DNA sequences from 10 clones were analyzed for each sample. Pie charts represent the proportion of clones with G-to-A and C-to-T mutations for left-side matrices. The total number of independent clones is indicated in the center. The number of mutations is indicated on the periphery of the pie segment. ***P<0.005. The statistical significance for the frequency of G-to-A mutations was calculated by chi-square test. (C) Selective PCR amplification for cccDNA. The template DNA samples were serially diluted 1/5 and 1/25 and amplified by pol-f/pol-r or ccc-f/ccc-r primer set. P: pCSD3.5 DHBV replicon plasmid starting at 1010 copies per reaction. N: nuclear Hirt-extracted DNA from pCSD3.5 transfectant. S: NC-DNA from culture supernatant of pCSD3.5 transfectants. The nuclear Hirt-extracted DNA conta [file ppat.1003361.s003.tif]

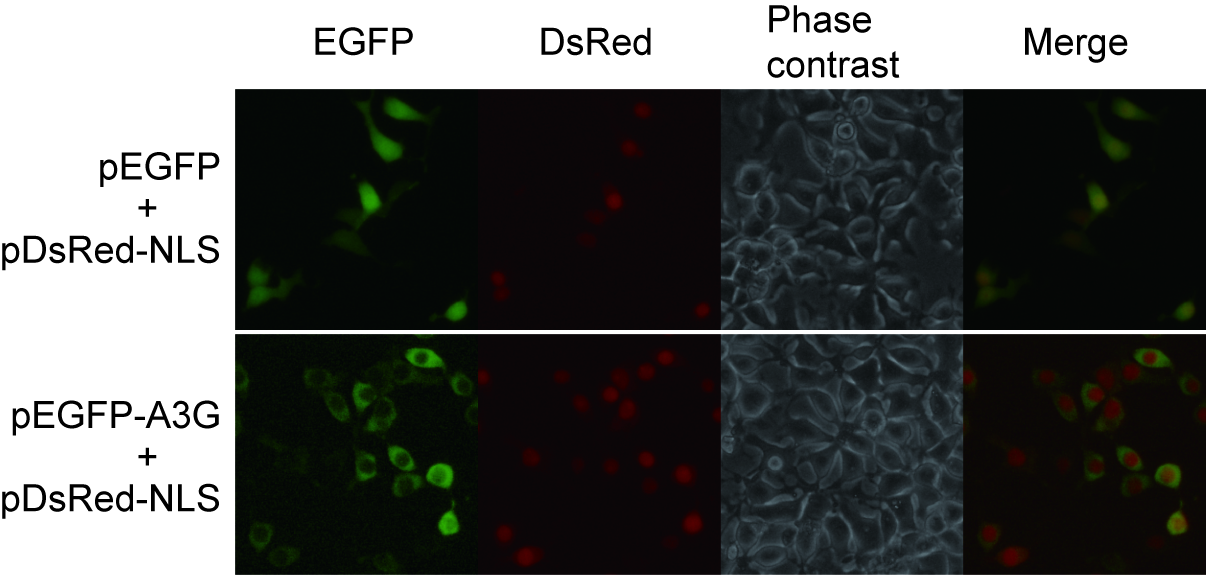

Supplement: Figure S4 — Intracellular localization of the A3G protein in LMH cells. Surface-deficient DHBV and EGFP-A3G or control EGFP vectors were used to transfect LMH cells. The nucleus was visualized with simultaneous expression of the DsRed-NLS protein. The EGFP protein was distributed in the nucleus and cytoplasm, whereas majority of GFP signals from the EGFP–A3G fusion protein come from cytoplasm. (TIF) [file ppat.1003361.s004.tif]

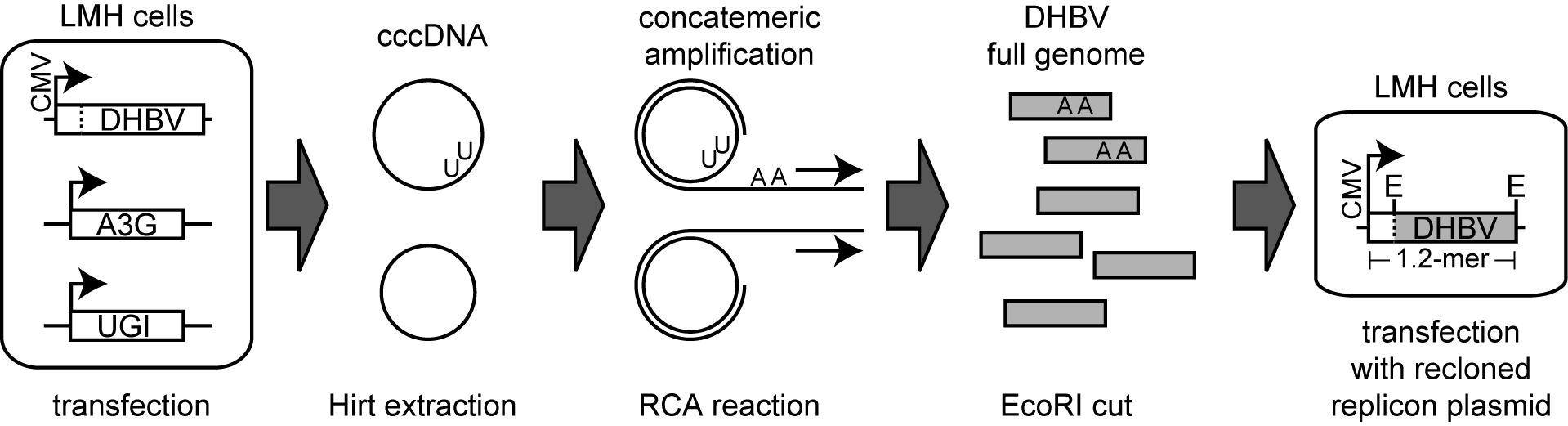

Supplement: Figure S5 — Experimental scheme for Figure 7 . The cccDNAs were purified from the cells 7 days after transfection and then treated with DpnI to digest any contaminating plasmids. The cccDNA was amplified by RCA and digested using EcoRI to produce 1 full-length copy of viral genomic DNA (Figure 7A). These EcoRI fragments were cloned into the replicon plasmid backbone (using the CMV promoter) to reconstruct the DHBV replicon plasmids. After transformation of reconstructed plasmids, 20 transformed and reconstructed E. coli clones were selected randomly from each sample. Twenty minipreps for each sample were prepared and DNA concentrations were estimated. From the 20 reconstructed replicon plasmids, 0.5 µg were taken, pooled, and used to transfect LMH cells without A3G or UGI vectors. Three days after transfection, NC-DNA was purified and quantified by qPCR (Figure 7B). For sequence analysis of reconstructed clones, 10 clones were randomly selected from the 20 reconstructed clones and result is shown in Figure 7C. (TIF) [file ppat.1003361.s005.tif]

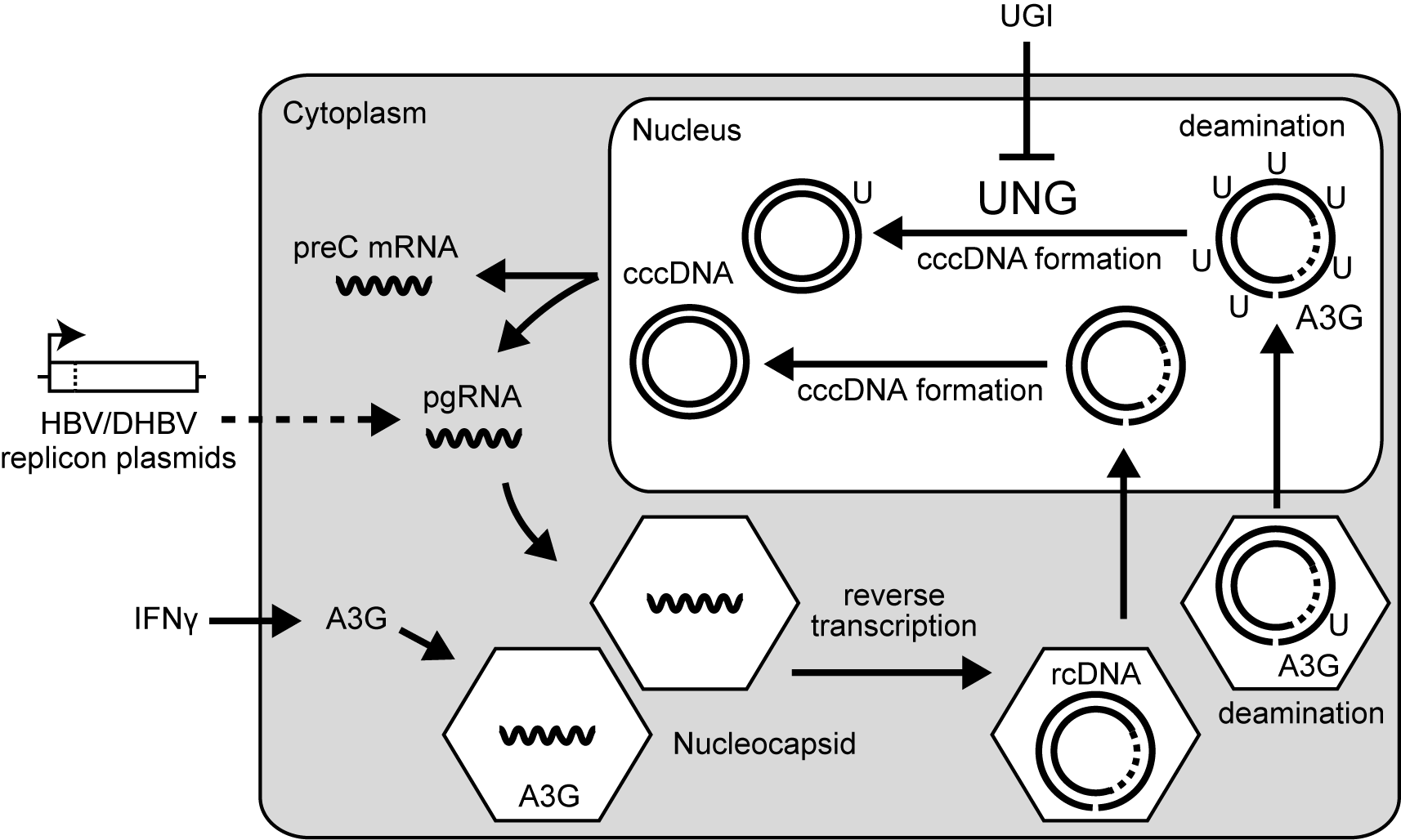

Supplement: Figure S6 — A proposed model to explain how UNG reduces uracil load on cccDNA. Intracellular viral lifecycle together with possible role of UNG. pgRNA is transcribed from cccDNA and the replicon plasmid when transfected. NC is assembled in the cytoplasm from core and P proteins together with pgRNA. In human hepatocytes, interferon induces APOBEC proteins such as A3G. A3G is encapsidated in a subset of NCs and induces hypermutation predominantly on the minus strand of rcDNA, resulting in G-to-A hypermutation. In addition, A3G inhibits minus strand DNA synthesis. After transportation into nucleus, additional hypermutation may be induced by A3G, and UNG repairs them during or after cccDNA formation. When UNG activity is inhibited by UGI, the extensive hypermutation remains in cccDNA, disrupting the genetic information for viral replication. Pre-C mRNA is transcribed from cccDNA but not from the replicon plasmid. When hypermutation does not affect any processes required for transcription, hypermutated transcripts such as pgRNA and pre-C mRNA are transcribed from hypermutated cccDNA. The hypermutated pgRNA may be encapsidated to enter a second viral lifecycle. (TIF) [file ppat.1003361.s006.tif]
