# Supplementary material for: Uracil DNA Glycosylase Counteracts APOBEC3G-Induced Hypermutation of Hepatitis B Viral Genomes: Excision Repair of Covalently Closed Circular DNA
Source: PLoS Pathog. 2013 May 16;9(5):e1003361. doi: 10.1371/journal.ppat.1003361 (PMC3656096; doi:10.1371/journal.ppat.1003361)
Supplement: Table S1 — List of plasmids used in this study. (PDF) [file ppat.1003361.s007.pdf]

Table S1. Vector list

Name	Description	Reference
pHBV1.5	A replication-competent HBV (adw2) plasmid, in which the HBV pgRNA is driven by endogenous HBV promoter.	[40][41]
pPB	A replication-competent HBV (adw R9) plasmid, in which the HBV pgRNA is driven by CMV promoter.	[44]
pCSD3.5	A replication-competent DHBV (DHBV3) plasmid, in which the DHBV pgRNA is driven by CMV promoter.	[49]
pCSD3.5ΔS	Three point mutations were introduced in pCSD3.5 to create in-frame stop codons at positions 1327, 1346, and 1349 in the surface protein ORF [49].	This study
pCSD3.5ΔP	A BamHI fragment (210bp) was deleted from pCSD3.5, resulting in desruption of functional P gene.	This study
pIRES-UGI	An UGI expression vector kindly gifted by Dr. Neuberger.	[51]
UGI-pFERp	An open reading frame of UGI was amplified by PCR with 5'-CTGAATTCATGACAAATTTATCT-3' and 5'-CTGGATCC GCTAACATTTTAATTTTA-3' primers and cloned into pFERp. After puromysin selection, this retrovirus vector stably expresses UGI-flag-ER chimeric protein.	This study
pFERp	AID cassette of pFB-AIDER-ires-puro retrovirus vector [38, 71] was replaced with a synthetic DNA linker containing multi-cloning site to make a mock retrovirus vector and designated as pFERp.	This study
mock-pFERp	Two oligonucleotides (5'-AATTCTAGCACCATGGCTAAG-3', 5'-GATCCTTAGCCATGGTGCTAG-3') containing an in-frame ATG were hybridized and inserted in an EcoRI/BamHI site of the pFERp retroviral vector. After puromysin selection, this retrovirus vector stably expresses flag-ER chimeric protein.	This study
pFLAG-GFP	A GFP2 ORF fragment from pGFP2N3 (Biosignal Packard) is inserted into pCMV3Tag2B (Invitrogen).	This study
pFLAG-A3G	Human A3G cDNA fragment amplified by PCR with 5'-AGGAATTCATGAAGCCTCACTT-3' and 5'-CCCCTCGA GCTAGTTTTCCTGATTCTGGA-3' primers was cloned into pcDNA3Tag1A (Invitrogen) and pEGFP-C2 (Clontech).	This study
pEGFP-A3G	see pFlag-A3G	This study
pcDNA3/HA-A3G	kindly gifted by Dr.Takaori.	[72]
pcDNA3/HA-E67QE259Q	A catalytic-inactive mutant A3G vector kindly gifted by Dr.Takaori.	[72]
pGFP-UNG2	Human UNG2 ORF amplified by PCR with 5'-TCGAAT TCATGATCGGCCAGAAGACG-3' and 5'-TTTTCTCGAGT CACAGCTCCTTCCA-3' primers was cloned into pEGFP-C2.	This study
pDsRed-NLS	Two copies of synthetic DNAs (hybridized oligonucleotides, 5'-TCGAGATCCAAAAAAGAAGAGAAAGGTACTTCG-3' and 5'-AATTCGAAGTACCTTTCTCTTCTTTTTTGGATC-3', or 5'-GATCCAAAAAAGAAGAGAAAGGTAG-3' and 5'-GATCTACCTTTCTCTTCTTTTTTG-3') encoding SV40 nuclear localization signal (NLS) was cloned into pDsRed monomer-C1 (Clontech).	This study

71. Nagaoka H, Ito S, Muramatsu M, Nakata M, Honjo T (2005) DNA cleavage in immunoglobulin somatic hypermutation depends on de novo protein synthesis but not on uracil DNA glycosylase. Proc Natl Acad Sci U S A 102: 2022-2027.

72. Shindo K, Takaori-Kondo A, Kobayashi M, Abudu A, Fukunaga K, et al. (2003) The enzymatic activity of CEM15/Apobec-3G is essential for the regulation of the infectivity of HIV-1 virion but not a sole determinant of its antiviral activity. J Biol Chem 278: 44412-44416.
