# Supplementary material for: Uracil DNA Glycosylase Counteracts APOBEC3G-Induced Hypermutation of Hepatitis B Viral Genomes: Excision Repair of Covalently Closed Circular DNA
Source: PLoS Pathog. 2013 May 16;9(5):e1003361. doi: 10.1371/journal.ppat.1003361 (PMC3656096; doi:10.1371/journal.ppat.1003361)
Supplement: Table S2 — List of primers used in this study. (PDF) [file ppat.1003361.s008.pdf]

Table S2. Primer list

Name	Sequence (5'→3')
Mutation analysis	(Y = C or T, R = G or A)
HBV 1st fwd	CGGAAATATACATCGTTTCCAT
HBV 1st rev	AAGAGTYYTYTTATGTAAGACYTT
HBV 2nd fwd	ATGGCTGCTARGCTGTACTGCCAA
HBV 2nd rev	AAGTGCACACGGAYYGGCAGAT
DHBV P gene fwd (pol-f)	GCGGGCTCCCCTCTCCCAC
DHBV P gene rev (pol-r)	CTGGATGGGCCGTCAGCAGGATTATA
DHBV cccDNA fwd (ccc-f)	CCTGATTGGACGGCTTTTCC
DHBV cccDNA rev (ccc-r)	CATTCTGCCGGCAAGTTGATTTAA
DHBV full fwd	TGACTGTACCTTTGGTATGTACCA
DHBV full rev	GTAGACGTAAAGATACCTTG
neo-f	TTGGGTGGAGAGGCTATTCG
neo-r	GGCGATACCGTAAAGCACGA
RCA	(* = modified with phosphorothioate)
DHBV179RCAf	ATTGAAGCAATCAC*T*
DHBV1069RCAf	CGAAGAAGATCAAA*A*
DHBV1827RCAf	CACCGAAATCAGAT*T*
DHBV2610RCAf	TTGGTATGTACCAT*T*
DHBV1RCAr	TTTCAAATGAGCAT*G*
DHBV786RCAr	CAGGAATTTGATGT*T*
DHBV1587RCAr	AGCTTTCCTAAAAT*A*
DHBV2330RCAr	TCTGATAACGTTTG*T*
qPCR	
HBV fwd	GAATTGATGACTCTAGCTACCTG
HBV rev	GAAACCACAATAGTTGCCTGATC
DHBV fwd	CAGCCTCAGTGGACTCCCGA
DHBV rev	GGCACAGCTGGTTCCGTCTGG
A3A fwd	ATGGCATTGGAAGGCATAAG
A3A rev	CAAAGAAGGAACCAGGTCCA
A3B fwd	TTCGAGGCCAGGTGTATTTCA
A3B rev	CAGAGATGGTCAGGGTGACA
A3C fwd	CAACGATCGGAACGAAACTT
A3C rev	TATGTCGTCGCAGAACCAAG
A3D fwd	ACCCAAACGTCAGTCGAATC
A3D rev	GCTCAGCCAAGAATTTGGTC
A3F fwd	GAAACACAGTGGAGCGAATG
A3F rev	GAAATGGGGCTCTGATGAAAG
A3G fwd	GGTCAGAGGACGGCATGAGA
A3G rev	GCAGGACCCAGGTGTCATTG
A3H fwd	CCCGCCTGTACTACCACTGG
A3H rev	GGGTTGAAGGAAAGCGGTTT
AICDA fwd	AAATGTCCGCTGGGCTAAGG
AICDA rev	GGAGGAAGAGCAATTCCACGT
HPRT fwd	GCCCTGGCGTCGTGATTAGT
HPRT rev	CGAGCAAGACGTTCAGTCCTGTC
DHBV preC fwd	CCTGATTGGACGGCTTTTCC
DHBV preC rev	GTAGACGTAAAGATACCTTG
DHBV-specific primer for RT reaction	GTAATGCCACCAAAGCAATCTCC
